# The Amsterdam petunia germplasm collection: A tool in plant science

**DOI:** 10.3389/fpls.2023.1129724

**Published:** 2023-03-21

**Authors:** Pamela Strazzer, Bets Verbree, Mattijs Bliek, Ronald Koes, Francesca M. Quattrocchio

**Affiliations:** Plant Development and Genetics, Swammerdam Institute of Life Sciences, University of Amsterdam, Amsterdam, Netherlands

**Keywords:** petunia, germplasm collection, model system, Solanaceae, speciation, mutant lines

## Abstract

*Petunia hybrida* is a plant model system used by many researchers to investigate a broad range of biological questions. One of the reasons for the success of this organism as a lab model is the existence of numerous mutants, involved in a wide range of processes, and the ever-increasing size of this collection owing to a highly active and efficient transposon system. We report here on the origin of petunia-based research and describe the collection of petunia lines housed in the University of Amsterdam, where many of the existing genotypes are maintained.

## Introduction

A large petunia germplasm collection is maintained at the University of Amsterdam (UvA) in the Netherlands. For decades it has supplied biological material for fundamental research and provided the basis for applications in agriculture and beyond.

Petunia-based research started over a century ago (and at the University of Amsterdam some 70 years ago) (Saunders, 1910; Bianchi, 1959) with studies on flower shape and pigmentation, including chemical analyses of anthocyanin pigments and other flavonoids (Birkhofer et al., 1963a; Birkhofer et al., 1963b; Birkhofer et al., 1965; Ando et al., 1999) and the inbreeding of commercial varieties for genetic analyses. Since then, the petunia has proven to be a very suitable system for studying flower pigmentation and several other processes related to the development of petals, in particular cells in the petal epidermis. These specialized cells for displaying color to attract pollinators are vastly different from the underlying mesophyll cells in their function, shape, and set of organelles (Li et al., 2021). In addition, the petunia has proved to be a suitable model for identifying genes and the mechanism involved in, for example, the regulation of gene expression, the definition of plant architecture, plant hormone biology, and plant speciation (see below).

## *Petunia* in the wild

*Petunia* species belong to the family of the Solanaceae, specifically the subfamily Petunieae (Petunioideae). The genus *Petunia* comprises 14 or 15 wild species, as well as a number of subspecies (Reck-Kortmann et al., 2014), that are endemic to South America (Wijsman et al., 1983; Ando and Hashimoto, 1995; Ando et al., 1995; Ando and Hashimoto, 1996; Ando and Hashimoto, 1998). The classification of species within the Solanaceae subfamilies has for a long time been based on flower morphology only; however, differences in morphological traits are often poorly correlated with genetic divergence (Ando et al., 2005; Kulcheski et al., 2006; Olmstead et al., 2008; Särkinen et al., 2013). *Calibrachoa*, for example, was until recently included in the *Petunia* genus (and is today still sold to consumers as “mini petunias”), but is now recognized as a separate genus because it has a different number of chromosomes. More recent extensive studies on Solanaceae classification are based on the sequence of a few (housekeeping) genes (Olmstead et al., 2008; Särkinen et al., 2013).

A transcriptome-wide phylogenetic analysis of these species revealed that *Petunia*, *Calibrachoa*, and *Fabiana* constitute a distinct clade separate from the other Petunieae. For instance, the genus *Brunfelsia* is more related to *Nierembergia*, *Leptoglossis*, *Bouchetia*, *Hunzikeria*, and *Plowmania* than to the *Petunia* clade (Wheeler et al., 2022).

**Figure 1:**
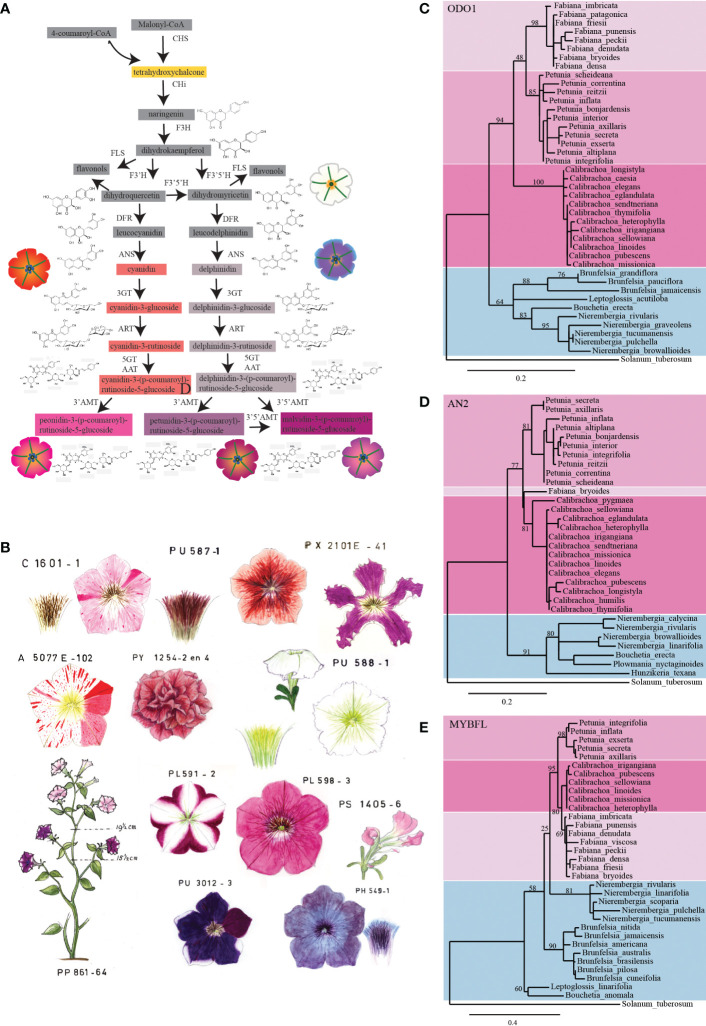
A *Petunia* collection uncovers the genetics of pollination syndrome. **(A)** Schematic representation of the anthocyanin biosynthetic pathway in *Petunia*. Intermediate products are shown in boxes; the color of the boxes corresponds to the color of the accumulated pigment intermediates. If these are colorless, the boxes are grey. The petunia flower drawings show the phenotype of different lines carrying mutations at specific steps of the pathway. The different colors of the petals are the result of the accumulation of specific intermediates of the pathway (dihydroflavonols, white; cyanidin derivatives, red; delphinidin derivatives, dull grey; peonidin derivatives, magenta; petunidin and malvidin derivatives, purple). Enzymes in the pathway are indicated in bold: CHS, chalcone synthase; CHI, chalcone isomerase; F3H, flavanone 3-hydroxylase; FLS, flavonol synthase; F3′H, flavonoid 3′-hydroxylase; F3′5′H, flavonoid 3′5′-hydroxylase; DFR, dihydroflavonol reductase; ANS, anthocyanidin synthase; 3GT, anthocyanidin 3-glucosyltransferase; ART, anthocyanidin 3-glucosyde rhamnosyltransferase; 5GT, anthocyanidin 5-glucosyltransferase; AAT, anthocyanidin 3-rutinoside acyltransferase; 3′AMT, anthocyanidin 3′ O-methyltransferase; 3′5′AMT, anthocyanidin 3′5′ O-methyltransferase. **(B)** Pictures of original historical petunia drawings. Collection of petunia mutants recorded as water-based drawings in the 1960s. The different mutant phenotypes show great variation in colors and shapes. Phylogenetic analysis of **(C)** ODO1, **(D)** AN2, and **(E)** MYBFL proteins from petunia and related species. In the ODO1 and AN2 trees, *Petunia*, *Calibrachoa*, and *Fabiana* form a distinct clade (as in Wheeler et al., 2022) separated from the other related species (i.e., from the genera *Brunfelsia*, *Nierembergia*, *Leptoglossis*, *Bouchetia*, *Hunzikeria*, and *Plowmania*). In the MYBFL tree, *Petunia*, *Calibrachoa*, and *Fabiana* also cluster together, but *Nierembergia* is a sister to this group. *PhODO*, *PhAN2*, and *PhMYBFL* transcripts were blasted against floral transcriptomes of different Petunieae species (Wheeler et al., 2022). The assembly of the reads into the predicted transcripts was performed with *de novo* assembler Trinity. The trees are built by maximum likelihood, after curation of the alignments with the G-BLOCKS tool and then rendered with TREEDYN using the online tools at http://www.Phylogeny.fr. Branch support is calculated on the basis of 300 bootstraps and indicated as a percentage. The protein sequences from which these trees were generated are reported in **Supplementary Table S2**.

The Smith Group at the University of Colorado, in collaboration with de Freitas from the Universidade Federal do Rio Grande do Sul in Brazil, carried out transcriptomic analyses across the Petunieae subfamily to investigate the relationship between floral anthocyanin variation and changes in gene expression (Ng et al., 2018). Their sampling comprised 72 species, including *Petunia* and other Petunieae that accumulate anthocyanins in the flower. Comparative methods highlighted that evolution of anthocyanin pigmentation in flowers occurred through sequential gain and loss of the activity of the two hydroxylating enzymes that shift the production from pelargonidin- to cyanidin (F3′H)-based anthocyanins and from cyanidin- to delphinidin (F3′5′H)-based anthocyanins (see **Figure 1A**).

Whereas Solanaceae are widely distributed across all continents (with the exception of Antarctica), Petunieae are found in Central and South America (including Patagonia). *Petunia* species are typically found in the tropical and subtropical areas of the South American continent (Chen et al., 2007). The most widely distributed *Petunia* species are *Petunia axillaris* and *Petunia integrifolia*, while other species, such as *P. exserta*, *P. bajeensis*, *P. bonjardinensis*, *P. mantiqueirensis*, *P. reitzii*, *P. saxicola*, and *P. secreta*, are found in very specific habitats only.

Distinct *Petunia* species display a remarkable diversity in plant size and shape and, most noticeably, in color and morphology of flowers. For example, species of the *Petunia axillaris* clade bear flowers with long tubes and white scented petals that are pollinated by hawkmoths. Species of the *P. inflata* clade instead have flowers with a short and wide tube, and violet non-scented petals that are pollinated by bees. Finally, another very different phenotype is shown by *P. exserta*, which has flowers with red petals that are pollinated by hummingbirds (Stuurman et al., 2004; Venail et al., 2010; Dell’Olivo et al., 2011; Hermann et al., 2015). Distinct species, even in places where they occur side by side (sympatric), remain genetically separated, as they are visited by different animals (Stuurman et al., 2004; Venail et al., 2010; Dell’Olivo et al., 2011; Hermann et al., 2015). Manual cross-pollination of natural *Petunia* species is however possible. The first such interspecific crosses were made in the early 19th century and gave rise to *Petunia hybrida*, or the garden petunia (Bailey, 1867; Wijsman et al., 1983). Over the next 200 years *P. hybrida* varieties were crossed numerous times with new accessions of wild species, and the genome of current *P. hybrida* varieties (2n = 14) is a mixture of multiple parental genomes (each 2n = 14) (Koes et al., 1987; Quattrocchio et al., 1999; Bombarely et al., 2016). The enormous variation between *P. hybrida* varieties stems from the introgression of mutant alleles from wild species and new mutations that arose during breeding.

## The start of a petunia germplasm collection

**Figure 2:**
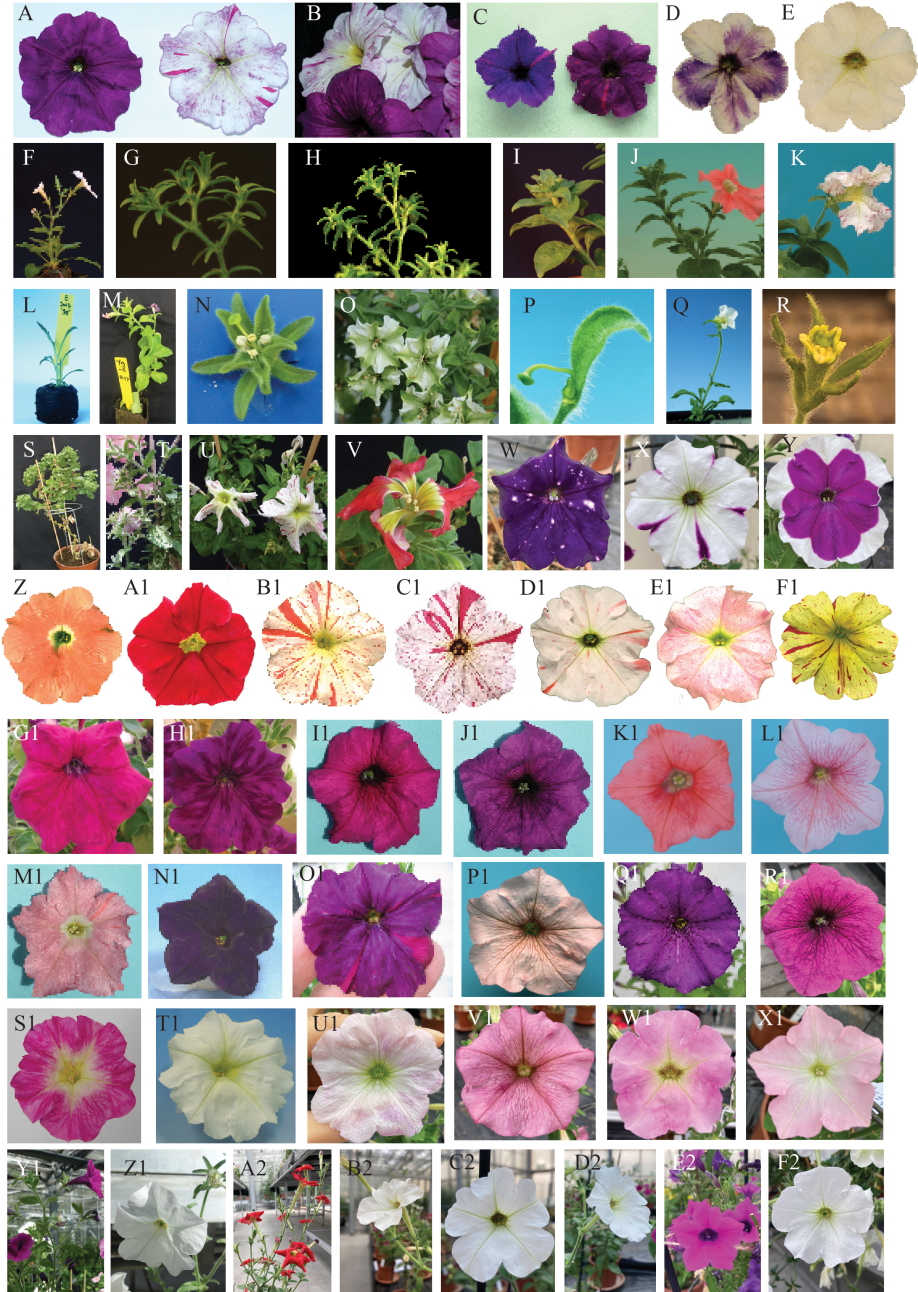
Phenotypes of a sample of different petunia lines from the Amsterdam collection.**(A)** Fading of flower pigmentation in the hybrid V74 × R149: on the right an *fa* mutant (pigmentation is stable) and on the left an *FA* wild type (Passeri et al., 2016). **(B)** A collection of flowers of different ages from a fading plant of the hybrid in **(A, C)** Transposon-induced mutation in a *PH* gene impairs vacuolar hyper-acidification and results in bluish petals. The more reddish spots are due to the excision of the transposon and restoration of hyper-acidification. On the right a *ph* mutant in an *FL* (flavonol accumulating) background and on the left the same *ph* mutation in an *fl* (no flavonols) background. **(D)** Flowers from a transgenic *AN2*-overexpressing line (Quattrocchio et al., 2013). **(E)** The untransformed host (line W115). **(F)** A wild-type inflorescence of the W138 petunia line. **(G)** Mutant in the flower meristem identity gene *ALF* (Souer et al., 2008) in a W138 background. **(H)** Mutant inflorescence for the flower meristem identity gene *DOT* (Souer et al., 2008) in a W138 background. **(I)** Mutant inflorescence in an *evergreen* (*evg*) plant. *EVERGREEN* encodes a WOX protein crucial in the separation of floral meristems from inflorescence meristem. In the mutant the inflorescence has a fasciated phenotype. **(J)** Mutant for the *VEGGIE* gene (Castel et al., 2010), in which flower identity determination is delayed, resulting in a series of bracts (instead of two) preceding the flower on the inflorescence. **(K)** The transposon-induced *hermit* mutant (Castel et al., 2010). **(L)** Transgenic line P7017 containing a *35S:NAM-vp16* (Souer et al., 1996). **(M)** Mutant in which the sympodial meristem is transformed into a vegetative meristem. **(N)** A strong mutant allele of the homeotic gene *GREENPETAL (GP).* The petals are fully transformed into sepals (Halfter et al., 1994; Vandenbussche et al., 2004). **(O)** A weak mutant allele of *GP*; the petals are only partially transformed into sepals. **(P)** Flower of a *floozy* (*flz*) (Tobeña-Santamaria et al., 2002) plant. *FLOOZY* encodes an enzyme involved in auxin synthesis. **(Q)** A *35S:DOT* (Souer et al., 2008) transgenic plant. The ectopic expression of this inflorescence identity gene results in very early flowering, changes the inflorescence in a terminal flower, and transforms leaf and sepal epidermal cells into petal epidermal cells. **(R)** Flower from a mutant for the gene *BLIND* (*BL*) (Cartolano et al., 2007) encoding a microRNA that regulates spatial expression of C-class homeotic genes in the flower. **(S)** Mutant with crinkled leaves, line P2036 (in the W138 background). **(T)** Unstable leaf mutant P2032. Several mutants with such leaf phenotype are often found among the progeny of W138. **(U)** Mutant P2056, a *choripetala Suzanne* (Vandenbussche et al., 2009) (*chsu*) mutant in the W138 background. The *CHORIPETALA SUZANNE* gene is involved in petal primordia fusion. **(V)** Mutant P2058 in the W138 background. A strong *chsu* allele gives “exploded” flowers. **(W)** Flower of the hybrid commercial genotype D2028. This, and the genotypes in X and Y, show pigmentation patterns due to suppression of *CHS* gene expression (Morita et al., 2012). **(X)** Flower of the hybrid commercial genotype E2011. **(Y)** Flower of the hybrid commercial genotype E2010. **(Z)** Flower of the inbred line R27, which accumulates cyanidin (*hf1*, *rt* mutant missing F3′5′H and rhamnosyl transferase activity). **(A1)** Flower of the inbred line R176 (originating from a reversion of the unstable *an1* allele in line W138). **(B1)** Flower of the inbred line W138. In this line, high transposon activity is maintained by selection, and in the progeny of this genotype new mutations continuously and spontaneously appear. **(C1)** Flower containing the *an1* mutable allele of W138 in a peonidin-accumulating background. **(D1)** Flower of a mutant for the *AN3* (van Houwelingen et al., 1998) locus encoding the F3H enzyme (see **Figure 1C**). The loss of activity of F3H results in a nearly white corolla as the petunia dihydroflavonol 4-reductase (DFR) cannot convert monohydroxylated anthocyanin precursors. This line is registered as W59. **(E1)** A weak allele of the *AN3* gene somehow results in low accumulation of anthocyanin. **(F1)** The flower of a hybrid of a carotenoid petunia line and the W138 line. **(G1)** Flower of a *PH5* wild-type plant (Verweij et al., 2008). **(H1)** Flower of a *ph5* mutant isogenic to the wild type in G1. **(I1)** Flower of the inbred line obtained for the cross M1 × V30 (Magenta 1 × Violet 30). **(J1)** Flower of a *ph4* (Quattrocchio et al., 2006) mutant generated by CRISPR-Cas9 technology in the hybrid M1 × V30. **(K1)** The R143 line contains a mutation at the *PH3* (Verweij et al., 2016) locus caused by the complete deletion of the gene. Because this mutation causes female sterility, the line can be maintained only by crossing heterozygous plants. Presented here is the flower of a WT plant arising from such a cross. **(L1)** A flower from a R143 mutant (*ph3*) plant. **(M1)** Flower from the R159 line carrying an unstable mutation (see the reddish reversion sectors) in the *PH5* locus. **(N1)** Flower of the inbred line V26 which carries a mutation in the *PH2* locus. **(O1)** Flower from an unstable *ph4* mutant in a malvidin-accumulating background. **(P1)** Flower of the line R153 containing a weak mutant allele of the *AN1* gene, also called *PH6* (Spelt et al., 2000), as this mutation affects only vacuolar acidification, without diminishing anthocyanin accumulation. The *ph6* allele is unstable, as shown by the reddish reversion sectors. **(Q1)** Flower of the inbred line V64, a stable *ph4* mutant. **(R1)** Flower of the inbred line V74, another stable *ph4* mutant. **(S1)** Flower of the M1 (Magenta 1) inbred line, which accumulates peonidin and carries a mutation in the *HF1* gene encoding the F3′5′H enzyme. (**T1**) Flower of the inbred line W225 carrying a stable mutant allele of the *AN1* gene. This allele carries a footprint originated from the excision of the *dTPH1* copy in the *an1* allele of W138. (**U1**) Flower of the inbred line W59 containing a mutant allele of the *AN2* gene. This allele is characterized by a 4bp insertion in the coding region, probably the footprint of a transposon that visited the locus. **(V1)** Flower of E2015, a hybrid of V63 (*ph4*) and R163 (*ph5*). **(W1)** Flower of the inbred line M61. **(X1)** Flower of the inbred line W80, which carries a mutation in the *AN6* locus encoding the enzyme DFR (Beld et al., 1989). **(Y1)** The wild-type accession *Petunia inflata* registered in the collection as S6. **(Z1)** The wild-type accession *Petunia axillaris N* registered in the collection as S26. **(A2)** The wild-type accession *Petunia exserta* registered in the collection as S25. **(B2)** The wild-type accession *Petunia parodii* registered in the collection as S8. **(C2)** The wild-type accession *Petunia axillaris* registered in the collection as S21. **(D2)** The wild-type accession *Petunia axillaris* registered in the collection as S2. **(E2)** The wild-type accession *Petunia integrifolia* registered in the collection as S20. **(F2)** The wild-type accession *Petunia axillaris P* registered in the collection as S21.

Over the years a collection of pure-breeding *P. hybrida* accessions has been generated from a plethora of (unrelated) commercial accessions. Classical genetic analysis of these lines identified a wealth of loci that determine the color of petals, anthers, and leaves, or various aspects of plant development (Wiering, 1974; Cornu and Maizonnier, 1983; de Vlaming et al., 1984), see some examples in **Figures 2A–F2**.

On several occasions, new mutants arose that displayed genetic instability (e.g., **Figure 2B1–D1**), frequently reverting to the wild type in somatic and sporogenic tissues, which is typical of transposon insertions (Malinowski, 1935; Cornu, 1977). In the 1970s the red-flowering line R27, which was inbred from the commercial Roter Vogel, produced mutant progeny with white petals with numerous red spots due to a new unstable mutation in the *ANTHOCYANIN1* locus. This was maintained in the line White 138 (W138) (Bianchi et al., 1978; Doodeman et al., 1984). Progeny of W138 produced unstable mutations at other loci at high frequency (Doodeman et al., 1984; van Houwelingen et al., 1998).

Molecular analyses revealed that the large majority of these unstable mutations resulted from insertions of a small (284-bp) non-autonomous transposon of the non autonomous transposon of the hobo, Activator, Tam3 (hAT) family named d*TPH1* (Gerats et al., 1990; van Houwelingen et al., 1998; Spelt et al., 2000). This paved the way to molecularly identify a wealth of new genes involved in, for example, flower pigmentation (de Vetten et al., 1997; Quattrocchio et al., 1999; Spelt et al., 2000; Quattrocchio et al., 2006; Verweij et al., 2008; Verweij et al., 2016) and plant development (Souer et al., 1996; Tobeña-Santamaria et al., 2002; Cartolano et al., 2007; Rebocho et al., 2008; Castel et al., 2010) *via* d*TPH1*-tagged mutant alleles, and to obtain mutants of genes whose sequence was known but for which no indication of function was available (Koes et al., 1995; Vandenbussche et al., 2008).

Over the years, the spontaneous appearance of transposon-induced mutations, together with ethyl methanesulfonate (EMS) mutagenesis and more recently the CRISPR-Cas approach, has resulted in a colorful collection of novel lines carrying mutations in genes involved in many different processes.

In the early years of this petunia collection, the phenotypes of established lines and newly emerged mutants were recorded by means of water-based drawings (**Figure 1B**), as color photography poorly reproduced the true colors. Recently, these drawings inspired the artist Christian Herren (Herren, 2021a; Herren, 2021b) to produce different works illustrating the use of the small garden petunia to address scientific questions. Later, watercolor paintings were replaced by digital pictures that record the phenotype of each mutant/line (**Figure 2**).

## Regulation of pigmentation and related processes in *Petunia*

The ability to identify and isolate new mutations is largely affected by how difficult it is to spot the new phenotype. Among the new mutants emerging in the collection, the easiest to spot are those heavily affecting the plant architecture and those affecting the biosynthetic pathway of anthocyanins and co-pigments such as flavonols (both structural and regulatory genes) (van Houwelingen et al., 1998) (see **Figure 1A**); the hyper-acidification of the lumen of the vacuoles where the pigments are stored (also structural and regulatory genes) (Spelt et al., 2000; Quattrocchio et al., 2006; Verweij et al., 2008; Faraco et al., 2014; Verweij et al., 2016); the formation of additional vacuoles (Faraco et al., 2017); and the shape and dimension of the cells (Li et al., 2021). Many genes involved in these processes were identified through mutants that appeared spontaneously in progeny of W138 and derived lines.

Compared with other systems in which pigmentation and related phenomena have been studied, *Petunia* offers the most complete description of the genetics behind the coloration of plant tissues by anthocyanins. This includes the regulation of the biosynthesis of these pigments, the differentiation of cells in petal epidermis, where coloration is displayed, and the contribution of several other factors to the final color. Other species in which pigmentation was studied, including bright-colored flowers such as snapdragons (Albert et al., 2021), gerberas (Deng et al., 2014), lilies (Yamagishi, 2020), and orchids (Liang et al., 2020), and other pigmented organs such as oranges (Huang et al., 2018), apples (Chagné et al., 2013), perilla (Jiang et al., 2020), and lychees (Lai et al., 2019), have a poor set of genetics tools, lack a good transposon system, or are not easy to transform. In others with excellent genetic tools (e.g., *Arabidopsis* and tomatoes), anthocyanin production is limited to small parts of the plant under stress conditions (Li and Strid, 2005; Li et al., 2018).

The hyper-acidification mechanism of vacuoles in specialized cells, such as the epidermis of petals (Verweij et al., 2008; Faraco et al., 2014) and the flesh of fruits (Strazzer et al., 2019), was first recognized in petunias because of the shift in color in the mutant petals, and was shown to require activity of two, until then unknown, types of P-type ATPases. It was found that the same mechanism operates in other species, in petals (e.g., rose petals) or other tissues such as fruit (e.g., in citrus and grapes) (Wang et al., 2014; Cavallini et al., 2015; Li et al., 2016; Amato et al., 2017). Remarkably, this was not first discovered in *Arabidopsis*, the most popular plant model in which genomic tools have been available for longer. The reason for this is that the gene for one of the two pumps was lost from the *Arabidopsis* genome (Li et al., 2016). Similarly, *Arabidopsis* is not useful for studying the mechanism for the formation of acidic additional vacuoles (vacuolinos) in specialized tissues (Faraco et al., 2017) because the small GTPase RAB5a, a key player in the formation of these organelles, is absent from the RAB5 subgroup of Brassicaceae (Li et al., 2021).

Studies on the production of other pigments, such as carotenoids, are ongoing in petunias. These are partly driven by the ornamental market, which prefers rare yellow/orange colors; therefore, new yellow inbred lines containing highly active transposons are being generated (e.g., **Figure 2F1**).

This all is facilitated by the brightly colored flowers of petunias, which are sufficiently large (3–7 cm in diameter depending on the line) that it is easy to spot mutations affecting petal color and to make molecular and biochemical studies very manageable.

## *Petunia* unravels the evolution of pollination syndrome

Efficient reproduction is the key to success for species in the struggle for survival. Changes in reproductive strategy result in genetic isolation and possibly in the appearance of a new species. The pool of traits that determine the chosen strategy of a plant species and, when needed, its interaction with pollinating animals (mostly insects or birds) is known as a pollination syndrome (Fenster et al., 2004; Rodrigues et al., 2018). The genetics behind the appearance of new pollination syndromes is the key to plant evolution biology and has been studied in several species. *Petunia* is represented in the wild by several species and subspecies that are genetically isolated in nature, but still produce viable seeds when manually pollinated. This allows for the generation of biological material to reconstruct the events that led to the appearance of new pollination syndromes and consequently new species (Hermann and Kuhlemeier, 2011; Turchetto et al., 2014).

Changes in the traits constituting the pollination syndrome of a species result in a new pollination strategy. The shape, color, and scent of the flower, as well as the amount of nectar and its composition, are the main traits involved (Klahre et al., 2011; Rodrigues et al., 2018). The identification of crucial mutations that lead to a novel pollination syndrome helps reconstruct the evolution of the distinct species in the *Petunia* genus, providing insights into the molecular mechanism of speciation. Mutations in the anthocyanin MYB regulator AN2 accompanied the appearance of the white species (*P. axillaris* subspecies). However, molecular analysis of the *an2* alleles in the white species indicates not that the loss of AN2 activity was initially responsible for the separation of the white lines, but rather that it contributed to a reinforcement mechanism (Quattrocchio et al., 1999).

Another MYB (MYB-FL) regulating the synthesis of flavonol co-pigments was shown to be directly related to the shift from bee pollination (in the purple-flowering *P. inflata*) to moth pollination through the acquisition of high expression (white *P. axillaris*) and then again to bird pollination by loss of activity (red *P. exserta*) (Sheehan et al., 2016). Recent evidence shows that the shift from white-lowering petunias to the red-flowering *P. exserta* was the result of a change in the expression domain for the *AN2* paralog *DPL* (Deep Purple), accompanied by reprogramming of the expression of hydroxylating genes and down-regulation of anthocyanin acyltransferase (Berardi et al., 2021).

The biochemistry and genetics of the production and release of fragrance in flowers, another component of the plant–pollinator interaction, is also most extensively studied in petunias (Muhlemann et al., 2014; Adebesin et al., 2017). It has been shown that two main loci are responsible for the acquisition or loss of scent in the transition between different pollination syndromes (Klahre et al., 2011).

Thanks to the brevity of their evolutionary separation, the process that generated the different wild petunia accessions can be relatively easily reconstructed. Using the data set published by Wheeler et al. (2022), we built the phylogeny of *Petunia* and related species based on the main speciation genes *ODO1* (Amrad et al., 2016), *AN2* (Quattrocchio et al., 1999), and *MYB-FL* (Sheehan et al., 2016). The result was an outline of the phylogeny of *Petunia* and closely related species based on their chosen pollinators (**Figures 1C–E**). This shows that, although these three genes did not evolve completely synchronously, the clade containing *Petunia*, *Calibrachoa*, and *Fabiana* is in all trees well separated from other closely related Petunieae. *Petunia* is moreover equally related to *Calibrachoa* and *Fabiana*, although its morphological similarity to the latter is clearly much less pronounced. The three genera are highly related but still clearly circumscribed (indicated in **Figures 1C–E** by the different color of the background), as reflected by the current taxonomy (Ng and Smith, 2016). They offer biological material to study whether the same mechanisms were adopted in the separation of white versus colored *Calibrachoa, Fabiana*, and *Petunia*, as well as scenting from non-scenting populations. The separation of different species within the *Petunia* genus must have occurred “very recently”, as supported by the fact that it is possible to obtain viable fertile plants from manual interspecific crosses (Yarahmadov et al., 2020), whereas hybrids of *Petunia* and *Calibrachoa* are rarely successful and the progeny is not fertile, which is to be expected given the differing chromosome sets of the two species.

## *Petunia* as model system in the study of different biological processes

Here, we give a succinct overview of some of the fields of research, outside pigmentation genetics, in which petunias have been the system of choice, reporting the most relevant discoveries that these studies generated.

An attempt to change the amount of anthocyanin in petals by expressing antisense or sense CHALCONE SYNTHASE (CHS) transgenes in petunias in the 1990s yielded flowers with intriguing pigmentation patterns on their petals (see **Figures 2W–Y**) and the discovery of RNA interference (RNAi) (van der Krol et al., 1988). This phenomenon, at the time not yet called RNAi, turned out to regulate a variety of processes in plants and animals (Han, 2018; Hung and Slotkin, 2021). The knockdown of single or groups of genes has found a multitude of applications in research (Matthew, 2004; Curtis and Nardulli, 2009) and medicine (Grimm and Kay, 2007). The finding that double-strand RNA triggers gene silencing through RNAi resulted the awarding of the 2006 Nobel Prize in Physiology or Medicine to Craig Mello and Andrew Fire (Zamore, 2006). Today this technology is, among techniques, the basis of advanced strategies for the treatment of AIDS development in HIV-positive patients (Swamy et al., 2016).

Distinct aspects of plant hormone synthesis and transport and their effect on plant physiology and development were discovered in petunias. A mutant with flowers lacking all organs except pistil and carpels (*floozy* mutant; **Figure 2P**) isolated in a W138 background revealed that flavin mono-oxygenase regulates the development of flower organs and leaves, affecting local auxins synthesis (Tobeña-Santamaria et al., 2002). The *dad* mutants described by Snowden et al. are characterized by increased branching and define steps of the strigolactone biosynthetic pathway (Snowden et al., 2005; Simons et al., 2007). The study of the synthesis of brassinosteroids (Drummond et al., 2009) and their signaling pathway and sensing (Verhoef et al., 2013), as well as the discovery of the protein involved in the transport of strigolactones (Kretzschmar et al., 2012), were enabled by mutants affecting these processes in *Petunia*.

The symbiosis between *Petunia* plants and mycorrhizae has been an effective instrument for identifying genes involved in infection initiation, development, and the morphology of arbuscular fungi (Sekhara Reddy et al., 2007), allowing for the identification of genes controlling different steps in these processes (Rich et al., 2015).

Adventitious root formation is the basis of vegetative propagation, which is important in the horticultural and ornamental industry. Hormonal regulation and the effect of ammonium and iron on this process, as well as the induction of genes involved in hormone transport and response at the site of adventitious root emergence, have been extensively studied in petunias (Druege and Franken, 2019).

The study of plant pararetroviruses and the contribution of these and retrotransposon-related viruses to the evolution of genomes has used different virus–host systems, including the petunia vein-clearing virus (PVCV) (Richert-Pöggeler et al., 2021). This virus interferes with the pigmentation patterns generated by RNAi silencing of the *CHS* gene. A decrease in DNA methylation of PVCV loci correlates with poor maintenance of DNA methylation in proviral PVCV and the appearance of pigmentation in otherwise white petal regions of star-type bicolored petals, suggesting that the virus could act as a suppressor of post-transcriptional gene silencing (Kuriyama et al., 2020).

## The collection of *Petunia* lines in Amsterdam

The petunia lines generated over decades of petunia-based research are preserved at the University of Amsterdam along with a detailed record of their origin and genetic characteristics. One of the many advantages of such a system is that isogenic lines can be compared when exploring the effect of single genes on any kind of process. Indeed, for many mutants, perfectly isogenic wild-type lines are available.

Most pure-breeding petunia lines, except for a few (V26 and Mitchell/W115), are difficult to transform by leaf disk transformation; however, all hybrids of two unrelated pure lines can easily generate transgenics (Vandenbussche et al., 2016). Owing to the multitude of lines available, it is possible to generate transformable hybrids for use in virtually any experimental setup. From some hybrids of two pure lines (e.g., M1 × V30) a new transformable (almost homozygous) line has been generated by repeated self-crosses. In such a background, some mutations have been introduced by CRISPR-Cas technology (**Figure 2I1, J1**), creating a set of isogenic mutants and wild types to be used in transformation experiments.

The documentation for each individual plant in the collection records its origin (father and mother), when it was grown, the phenotype, the transgene (if applicable), and any other unusual characteristics. These records have been kept and updated since the 1970s.

The storage of seeds in dedicated stores where the humidity and temperature can be controlled is crucial for their longevity. However, the renewal of the stock for each line through germination and the production of new seeds every 2–5 years is necessary to avoid loss of genotypes.

Here, we report a catalog of the lines present in the collection, complete with a description of the genetic background and main characteristics of each genotype (see **Supplementary Table S1**).

Seeds are available in principle (if the line is not involved in current projects) on request and agreement of conditions of use. This can be arranged by sending an e-mail to f.quattrocchio@uva.nl. A small fee is applied to cover the costs of line maintenance and seed production.

## Conclusion

A germplasm collection for a model species widely used in different fields within experimental life sciences is a valuable resource, and its preservation (and increase in available lines) makes it attractive for an ever-growing range of applications. Because little labor is required to generate new mutations, this model has proved highly effective in the identification of novel pathways that are absent or were lost during domestication of some of the popular alternative model species (e.g., *Arabidopsis* and tomatoes). Furthermore, the ability to compare several model species is a priority in evolutionary developmental biology, and comparisons between *Arabidopsis* and *Petunia* have resulted in interesting discoveries on several occasions.

This collection has for several decades been used for education in practical classes and was recently described by the Faculty of Humanities of the University of Amsterdam as an ‘archive of imagination’ and ‘mental shortcut’ to common heritage and history.

## Author contributions

PS, FQ, and RK conceived the idea of publishing a catalog of the petunia collection, carried out a literature survey, collected material, and wrote the manuscript. MB mined the transcriptomic data of Petunieae and BV prepared the actual catalog of the lines (**Supplementary Table S1**). All authors contributed to the article and approved the submitted version.

## References

[B1] Adebesin F. Widhalm J. R. Boachon B. Lefèvre F. Pierman B. Lynch J. H.. (2017). Emission of volatile organic compounds from petunia flowers is facilitated by an ABC transporter. Sci. (1979). 356, 1386–1388. doi: 10.1126/SCIENCE.AAN0826/SUPPL_FILE/AAN0826_ADEBESIN_SM.PDF 28663500

[B2] Albert N. W. Butelli E. Moss S. M. A. Piazza P. Waite C. N. Schwinn K. E.. (2021). Discrete bHLH transcription factors play functionally overlapping roles in pigmentation patterning in flowers of *Antirrhinum majus* . New Phytol. 231, 849–863. doi: 10.1111/NPH.17142 33616943 PMC8248400

[B3] Amato A. Cavallini E. Zenoni S. Finezzo L. Begheldo M. Ruperti B.. (2017). A grapevine TTG2-like WRKY transcription factor is involved in regulating vacuolar transport and flavonoid biosynthesis. Front. Plant Sci. 7. doi: 10.3389/FPLS.2016.01979 PMC5214514 28105033

[B4] Amrad A. Moser M. Mandel T. de Vries M. Schuurink R. C. Freitas L.. (2016). Gain and loss of floral scent production through changes in structural genes during pollinator-mediated speciation. Curr. Biol. 26, 3303–3312. doi: 10.1016/J.CUB.2016.10.023 27916524

[B5] Ando T. Hashimoto G. (1995). *Petunia guarapuavensis* (Solanaceae): A new species from planalto of paraná and Santa catarina, Brazil. Brittonia 47, 328–334. doi: 10.2307/2807120

[B6] Ando T. Hashimoto G. (1996). A new Brazilian species of *Petunia* (Solanaceae) from interior Santa catarina and Rio grande do sul, Brazil. Brittonia 48, 217–223. doi: 10.2307/2807818

[B7] Ando T. Hashimoto G. (1998). Two new species of *Petunia* (Solanaceae) from southern Rio grande do sul, Brazil. Brittonia 50, 483–492. doi: 10.2307/2807758

[B8] Ando T. Kokubun H. Watanabe H. Tanaka N. Yukawa T. Hashimoto G.. (2005). Phylogenetic analysis of *Petunia sensu* jussieu (Solanaceae) using chloroplast DNA RFLP. Ann. Bot. 96, 289–297. doi: 10.1093/AOB/MCI177 15944177 PMC4246877

[B9] Ando T. Kurata M. Sasaki S. Ueda Y. Hashimoto G. Marchesi E. (1995). Comparative morphological studies on infraspecific taxa of *Petunia integrifolia* (Hook,) schinz et thell. (Solanaceae). J. Japanese. Bot. 70, 205–217.

[B10] Ando T. Saito N. Tatsuzawa F. Kakefuda T. Yamakage K. Ohtani E.. (1999). Floral anthocyanins in wild taxa of *Petunia* (Solanaceae). Biochem. Syst. Ecol. 27, 623–650. doi: 10.1016/S0305-1978(98)00080-5

[B11] Bailey L. H. (1867). The survival of the unlike: A collection of evolution essays suggested by the study of domestic plants. Nature 56, 493–493.

[B12] Beld M. Martin C. Huits H. Stuitje A. R. Gerats A. G. M. (1989). Flavonoid synthesis in *Petunia hybrida*: partial characterization of dihydroflavonol-4-reductase genes. Plant Mol. Biol. 13, 491–502. doi: 10.1007/BF00027309 2491667

[B13] Berardi A. E. Esfeld K. Jäggi L. Mandel T. Cannarozzi G. M. Kuhlemeier C. (2021). Complex evolution of novel red floral color in *Petunia* . Plant Cell 33, 2273–2295. doi: 10.1093/PLCELL/KOAB114 33871652 PMC8364234

[B14] Bianchi F. (1959). Onderzoek naar de erfelijkheid van de bloemvorm bij petunia (Amsterdam: University of Amsterdam), 1–84. Available at: https://www.boekwinkeltjes.nl/b/107741751/Onderzoek-naar-de-erfelijkheid-van-de-bloemvorm-bij-Petunia/.

[B15] Bianchi F. Cornelissen P. T. J. Gerats A. G. M. Hogervorst J. M. W. (1978). Regulation of gene action in *Petunia hybrida*: Unstable alleles of a gene for flower colour. Theor. Appl. Genet. 53, 157–167. doi: 10.1007/BF00273576 24309594

[B16] Birkhofer L. Kaiser C. Donike M. Koch W. (1965). Konstitution von acyl-anthocyanen. Z. Naturforschung. 20b, 424–428. doi: 10.1515/znb-1965-0504

[B17] Birkhofer L. Kaiser C. Donike M. Wolf D. (1963a). Acylierte anthocyane II cis-trans-Isomerie bei aacyl-anthocyanen. Z. Naturforschung. 18b, 631–634. doi: 10.1515/znb-1963-0807

[B18] Birkhofer L. Kaiser C. Koch W. Lange H. (1963b). Nicht acylierte anthocyane in blüten von *Petunia hybrida* . Z. Naturforschung. 18b, 367–370. doi: 10.1515/znb-1963-0504

[B19] Bombarely A. Moser M. Amrad A. Bao M. Bapaume L. Barry C. S.. (2016). Insight into the evolution of the solanaceae from the parental genomes of *Petunia hybrida* . Nat. Plants 2, 1–9. doi: 10.1038/NPLANTS.2016.74 27255838

[B20] Cartolano M. Castillo R. Efremova N. Kuckenberg M. Zethof J. Gerats T.. (2007). A conserved microRNA module exerts homeotic control over *Petunia hybrida* and *Antirrhinum majus* floral organ identity. Nat. Genet. 39, 901–905. doi: 10.1038/ng2056 17589508

[B21] Castel R. Kusters E. Koes R. (2010). Inflorescence development in petunia: through the maze of botanical terminology. J. Exp. Bot. 61, 2235–2246. doi: 10.1093/JXB/ERQ061 20308206

[B22] Cavallini E. Matus J. T. Finezzo L. Zenoni S. Loyola R. Guzzo F.. (2015). The phenylpropanoid pathway is controlled at different branches by a set of R2R3-MYB C2 repressors in grapevine. Plant Physiol. 167, 1448–1470. doi: 10.1104/PP.114.256172 25659381 PMC4378173

[B23] Chagné D. Lin-Wang K. Espley R. v. Volz R. K. How N. M. Rouse S.. (2013). An ancient duplication of apple MYB transcription factors is responsible for novel red fruit-flesh phenotypes. Plant Physiol. 161, 225–239. doi: 10.1104/PP.112.206771 23096157 PMC3532254

[B24] Chen S. Matsubara K. Omori T. Kokubun H. Kodama H. Watanabe H.. (2007). Phylogenetic analysis of the genus *Petunia* (Solanaceae) based on the sequence of the *Hf1* gene. J. Plant Res. 120, 385–397. doi: 10.1007/S10265-006-0070-Z/FIGURES/6 17353990

[B25] Cornu A. (1977). Systemes instables induits chez le petunia. Mutat. Research/Fundamental. Mol. Mech. Mutagenesis. 42, 235–248. doi: 10.1016/S0027-5107(77)80027-4

[B26] Cornu A. Maizonnier D. (1983). The genetics of *Petunia* . Plant Breed. Rev. 1, 11–58. doi: 10.1007/978-1-4684-8896-8_2

[B27] Curtis C. D. Nardulli A. M. (2009). Using RNA interference to study protein function. Methods Mol. Biol. 505, 187. doi: 10.1007/978-1-60327-575-0_11 19117146 PMC3900309

[B28] Dell’Olivo A. Hoballah M. E. Gübitz T. Kuhlemeier C. (2011). Isolation barriers between *Petunia axillaris* and *Petunia integrifolia* (Solanaceae). Evolution 65, 1979–1991. doi: 10.1111/J.1558-5646.2011.01279.X 21729053

[B29] Deng X. Bashandy H. Ainasoja M. Kontturi J. Pietiäinen M. Laitinen R. A. E.. (2014). Functional diversification of duplicated chalcone synthase genes in anthocyanin biosynthesis of *Gerbera hybrida* . New Phytol. 201, 1469–1483. doi: 10.1111/NPH.12610 24266452

[B30] de Vetten N. Quattrocchio F. Mol J. Koes R. (1997). The *an11* locus controlling flower pigmentation in petunia encodes a novel WD-repeat protein conserved in yeast, plants, and animals. Genes Dev. 11, 1422–1434. doi: 10.1101/GAD.11.11.1422 9192870

[B31] de Vlaming P. Gerats A. G. M. Wiering H. Wijsman H. J. W. Cornu A. Farcy E.. (1984). *Petunia hybrida*: A short description of the action of 91 genes, their origin and their map location. Plant Mol. Biol. Rep. 2, 21–42. doi: 10.1007/BF03015868

[B32] Doodeman M. Boersma E. A. Koomen W. Bianchi F. (1984). Genetic analysis of instability in *Petunia hybrida*: 1. a highly unstable mutation induced by a transposable element inserted at the *An1* locus for flower colour. Theor. Appl. Genet. 67, 345–355. doi: 10.1007/BF00272873 24258658

[B33] Druege U. Franken P. (2019). Petunia as model for elucidating adventitious root formation and mycorrhizal symbiosis: at the nexus of physiology, genetics, microbiology and horticulture. Physiol. Plant 165, 58–72. doi: 10.1111/PPL.12762 29774547 PMC7380035

[B34] Drummond R. S. M. Marcela Martínez-Sánchez N. Janssen B. J. Templeton K. R. Simons J. L. Quinn B. D.. (2009). *Petunia hybrida* CAROTENOID CLEAVAGE DIOXYGENASE7 is involved in the production of negative and positive branching signals in petunia. Plant Physiol. 151, 1867–1877. doi: 10.1104/PP.109.146720 19846541 PMC2785980

[B35] Faraco M. Li Y. Li S. Spelt C. di Sansebastiano G. Reale L.. (2017). A tonoplast P3B-ATPase mediates fusion of two types of vacuoles in petal cells. Cell Rep. 19, 2413–2422. doi: 10.1016/J.CELREP.2017.05.076 28636930

[B36] Faraco M. Spelt C. Bliek M. Verweij W. Hoshino A. Espen L.. (2014). Hyperacidification of vacuoles by the combined action of two different p-ATPases in the tonoplast determines flower color. Cell Rep. 6, 32–43. doi: 10.1016/J.CELREP.2013.12.009 24388746

[B37] Fenster C. B. Armbruster W. S. Wilson P. Dudash M. R. Thomson J. D. (2004). Pollination syndromes and floral specialization. Annu. Rev. Ecol. Evol. Syst. 35, 375–403. doi: 10.2307/annurev.ecolsys.34.011802.30000015

[B38] Gerats A. G. M. Huits H. Vrijlandt E. Marañ C. Souer E. Beld M. (1990). Molecular characterization of a nonautonomous transposable element (dTph1) of petunia. Plant Cell 2, 1121–1128. doi: 10.1105/TPC.2.11.1121 1967052 PMC159959

[B39] Grimm D. Kay M. A. (2007). RNAi and gene therapy: a mutual attraction. Hematol. Am. Soc. Hematol. Educ. Program, 473–481. doi: 10.1182/ASHEDUCATION-2007.1.473 18024667

[B40] Halfter U. Ali N. Stockhaus J. Ren L. Chua N. H. (1994). Ectopic expression of a single homeotic gene, the *Petunia* gene green petal, is sufficient to convert sepals to petaloid organs. EMBO J. 13, 1443. doi: 10.1002/J.1460-2075.1994.TB06398.X 7907980 PMC394962

[B41] Han H. (2018). RNA Interference to knock down gene expression. Methods Mol. Biol. 1706, 293–302. doi: 10.1007/978-1-4939-7471-9_16 29423805 PMC6743327

[B42] Hermann K. Klahre U. Venail J. Brandenburg A. Kuhlemeier C. (2015). The genetics of reproductive organ morphology in two *Petunia* species with contrasting pollination syndromes. Planta 241, 1241–1254. doi: 10.1007/S00425-015-2251-2 25656052

[B43] Hermann K. Kuhlemeier C. (2011). The genetic architecture of natural variation in flower morphology. Curr. Opin. Plant Biol. 14, 60–65. doi: 10.1016/J.PBI.2010.09.012 20934369

[B44] Herren C. (2021a) I Really wanna be with you. Available at: https://sandberg.nl/i-really-wanna-be-with-you.

[B45] Herren C. (2021b) To be rebuilt with the materials of your time. Available at: https://www.festivalofchoices.nl/christian-herren.html.

[B46] Huang D. Wang X. Tang Z. Yuan Y. Xu Y. He J.. (2018). Subfunctionalization of the *Ruby2-Ruby1* gene cluster during the domestication of citrus. Nat. Plants 4, 930–941. doi: 10.1038/S41477-018-0287-6 30374094

[B47] Hung Y. H. Slotkin R. K. (2021). The initiation of RNA interference (RNAi) in plants. Curr. Opin. Plant Biol. 61, 1–9. doi: 10.1016/J.PBI.2021.102014 33657510

[B48] Jiang T. Guo K. Liu L. Tian W. Xie X. Wen S.. (2020). Integrated transcriptomic and metabolomic data reveal the flavonoid biosynthesis metabolic pathway in *Perilla frutescens* (L.) leaves. Sci. Rep. 10, 1–11. doi: 10.1038/S41598-020-73274-Y 33004940 PMC7530993

[B49] Klahre U. Gurba A. Hermann K. Saxenhofer M. Bossolini E. Guerin P. M.. (2011). Pollinator choice in *Petunia* depends on two major genetic loci for floral scent production. Curr. Biol. 21, 730–739. doi: 10.1016/J.CUB.2011.03.059 21497087

[B50] Koes R. Souer E. van Houwelingen A. Mur L. Spelt C. Quattrocchio F.. (1995). Targeted gene inactivation in petunia by PCR-based selection of transposon insertion mutants. Proc. Natl. Acad. Sci. U.S.A. 92, 8149–8153. doi: 10.1073/PNAS.92.18.8149 7667260 PMC41113

[B51] Koes R. E. Spelt C. E. Mol J. N. M. Gerats A. G. M. (1987). The chalcone synthase multigene family of *Petunia hybrida* (V30): sequence homology, chromosomal localization and evolutionary aspects. Plant Mol. Biol. 10, 159–169. doi: 10.1007/BF00016153 24277501

[B52] Kretzschmar T. Kohlen W. Sasse J. Borghi L. Schlegel M. Bachelier J. B.. (2012). A petunia ABC protein controls strigolactone-dependent symbiotic signalling and branching. Nature 483, 341–344. doi: 10.1038/NATURE10873 22398443

[B53] Kulcheski F. R. Muschner V. C. Lorenz-Lemke A. P. Stehmann J. R. Bonatto S. L. Salzano F. M.. (2006). Molecular phylogenetic analysis of *Petunia* juss. (Solanaceae). Genetica 126, 3–14. doi: 10.1007/S10709-005-1427-2 16502081

[B54] Kuriyama K. Tabara M. Moriyama H. Kanazawa A. Koiwa H. Takahashi H.. (2020). Disturbance of floral colour pattern by activation of an endogenous pararetrovirus, petunia vein clearing virus, in aged petunia plants. Plant J. 103, 497–511. doi: 10.1111/TPJ.14728 32100385 PMC7496347

[B55] Lai B. Du L. N. Hu B. Wang D. Huang X. M. Zhao J. T.. (2019). Characterization of a novel litchi R2R3-MYB transcription factor that involves in anthocyanin biosynthesis and tissue acidification. BMC Plant Biol. 19, 1–13. doi: 10.1186/s12870-019-1658-5 30732564 PMC6367832

[B56] Li S. Cerri M. Strazzer P. Li Y. Spelt C. Bliek M.. (2021). An ancient RAB5 governs the formation of additional vacuoles and cell shape in petunia petals. Cell Rep. 36, 1–17. doi: 10.1016/J.CELREP.2021.109749 34592147

[B57] Li Y. Provenzano S. Bliek M. Spelt C. Appelhagen I. Machado de Faria L.. (2016). Evolution of tonoplast p-ATPase transporters involved in vacuolar acidification. New Phytol. 211, 1092–1107. doi: 10.1111/NPH.14008 27214749

[B58] Li S. Strid Å. (2005). Anthocyanin accumulation and changes in *CHS* and *PR-5* gene expression in *Arabidopsis thaliana* after removal of the inflorescence stem (decapitation). Plant Physiol. Biochem. 43, 521–525. doi: 10.1016/J.PLAPHY.2005.05.004 15993620

[B59] Li N. Wu H. Ding Q. Li H. Li Z. Ding J.. (2018). The heterologous expression of *Arabidopsis* PAP2 induces anthocyanin accumulation and inhibits plant growth in tomato. Funct. Integr. Genomics 18, 341–353. doi: 10.1007/S10142-018-0590-3 29372433

[B60] Liang C. Y. Rengasamy K. P. Huang L. M. Hsu C. C. Jeng M. F. Chen W. H.. (2020). Assessment of violet-blue color formation in *Phalaenopsis* orchids. BMC Plant Biol. 20, 1–16. doi: 10.1186/S12870-020-02402-7 32397954 PMC7218627

[B61] Malinowski E. (1935). Studies on unstable characters in petunia. i. the extreme flower types of the unstable race with mosaic color patterns. Genetics 20, 342–356. doi: 10.1093/GENETICS/20.4.342 17246763 PMC1208615

[B62] Matthew L. (2004). RNAi for plant functional genomics. Comp. Funct. Genomics 5, 240. doi: 10.1002/CFG.396 18629158 PMC2447448

[B63] Morita Y. Saito R. Ban Y. Tanikawa N. Kuchitsu K. Ando T.. (2012). Tandemly arranged chalcone synthase a genes contribute to the spatially regulated expression of siRNA and the natural bicolor floral phenotype in *Petunia hybrida* . Plant J. 70, 739–749. doi: 10.1111/J.1365-313X.2012.04908.X 22288551

[B64] Muhlemann J. K. Klempien A. Dudareva N. (2014). Floral volatiles: From biosynthesis to function. Plant Cell Environ. 37, 1936–1949. doi: 10.1111/PCE.12314 24588567

[B65] Ng J. Freitas L. B. Smith S. D. (2018). Stepwise evolution of floral pigmentation predicted by biochemical pathway structure. Evolution 72, 2792–2802. doi: 10.2307/48576790 30187462

[B66] Ng J. Smith S. D. (2016). Widespread flower color convergence in solanaceae *via* alternate biochemical pathways. New Phytol. 209, 407–417. doi: 10.1111/NPH.13576 26224118

[B67] Olmstead R. G. Bohs L. Migid H. A. Santiago-Valentin E. Garcia V. F. Collier S. M. (2008). A molecular phylogeny of the solanaceae. Taxon 57, 1159–1181. doi: 10.1002/TAX.574010

[B68] Passeri V. Koes R. Quattrocchio F. M. (2016). New challenges for the design of high value plant products: Stabilization of anthocyanins in plant vacuoles. Front. Plant Sci. 7. doi: 10.3389/FPLS.2016.00153 PMC4754442 26909096

[B69] Quattrocchio F. M. Spelt C. Koes R. (2013). Transgenes and protein localization: myths and legends. Trends Plant Sci. 18, 473–476. doi: 10.1016/J.TPLANTS.2013.07.003 23932488

[B70] Quattrocchio F. Verweij W. Kroon A. Spelt C. Mol J. Koes R. (2006). PH4 of petunia is an R2R3 MYB protein that activates vacuolar acidification through interactions with basic-helix-loop-helix transcription factors of the anthocyanin pathway. Plant Cell 18, 1274–1291. doi: 10.1105/tpc.105.034041 16603655 PMC1456866

[B71] Quattrocchio F. Wing J. van der Woude K. Souer E. de Vetten N. Joseph M.. (1999). Molecular analysis of the *anthocyanin2* gene of *Petunia* and its role in the evolution of flower color. Plant Cell 11, 1433–1444. doi: 10.1105/TPC.11.8.1433 10449578 PMC144295

[B72] Rebocho A. B. Bliek M. Kusters E. Castel R. Procissi A. Roobeek I.. (2008). Role of EVERGREEN in the development of the cymose petunia inflorescence. Dev. Cell 15, 437–447. doi: 10.1016/J.DEVCEL.2008.08.007 18804438

[B73] Reck-Kortmann M. Silva-Arias G. A. Segatto A. L. A. Mäder G. Bonatto S. L. de Freitas L. B. (2014). Multilocus phylogeny reconstruction: New insights into the evolutionary history of the genus *Petunia* . Mol. Phylogenet. Evol. 81, 19–28. doi: 10.1016/J.YMPEV.2014.08.022 25196589

[B74] Rich M. K. Schorderet M. Bapaume L. Falquet L. Morel P. Vandenbussche M.. (2015). The *Petunia* GRAS transcription factor ATA/RAM1 regulates symbiotic gene expression and fungal morphogenesis in arbuscular mycorrhiza. Plant Physiol. 168, 788–797. doi: 10.1104/PP.15.00310 25971550 PMC4741351

[B75] Richert-Pöggeler K. R. Vijverberg K. Alisawi O. Chofong G. N. Heslop-Harrison J. S. Schwarzacher T. (2021). Participation of multifunctional RNA in replication, recombination and regulation of endogenous plant pararetroviruses (EPRVs). Front. Plant Sci. 12. doi: 10.3389/FPLS.2021.689307/BIBTEX PMC8256270 34234799

[B76] Rodrigues D. M. Caballero-Villalobos L. Turchetto C. Jacques R. A. Kuhlemeier C. Freitas L. B. (2018). Do we truly understand pollination syndromes in *Petunia* as much as we suppose? AoB. Plants 10, 1–15. doi: 10.1093/AOBPLA/PLY057 PMC6202611 30386543

[B77] Särkinen T. Bohs L. Olmstead R. G. Knapp S. (2013). A phylogenetic framework for evolutionary study of the nightshades (Solanaceae): a dated 1000-tip tree. BMC Evol. Biol. 13, 1–15. doi: 10.1186/1471-2148-13-214 24283922 PMC3850475

[B78] Saunders E. R. (1910). Studies in the inheritance of doubleness in flowers: I. *Petunia* . J. Genet. 1, 57–69. doi: 10.1007/BF02981569/METRICS

[B79] Sekhara Reddy D. M. R. Schorderet M. Feller U. Reinhardt D. (2007). A petunia mutant affected in intracellular accommodation and morphogenesis of arbuscular mycorrhizal fungi. Plant J. 51, 739–750. doi: 10.1111/J.1365-313X.2007.03175.X 17573800

[B80] Sheehan H. Moser M. Klahre U. Esfeld K. Dell’olivo A. Mandel T.. (2016). MYB-FL controls gain and loss of floral UV absorbance, a key trait affecting pollinator preference and reproductive isolation. Nat. Genet. 48, 159–166. doi: 10.1038/NG.3462 26656847

[B81] Simons J. L. Napoli C. A. Janssen B. J. Plummer K. M. Snowden K. C. (2007). Analysis of the *DECREASED APICAL DOMINANCE* genes of petunia in the control of axillary branching. Plant Physiol. 143, 697–706. doi: 10.1104/PP.106.087957 17158589 PMC1803742

[B82] Snowden K. C. Simkin A. J. Janssen B. J. Templeton K. R. Loucas H. M. Simons J. L.. (2005). The decreased apical dominance1/*Petunia hybrida CAROTENOID CLEAVAGE DIOXYGENASE8* gene affects branch production and plays a role in leaf senescence, root growth, and flower development. Plant Cell 17, 746–759. doi: 10.1105/TPC.104.027714 15705953 PMC1069696

[B83] Souer E. Rebocho A. B. Bliek M. Kusters E. de Bruin R. A. M. Koes R. (2008). Patterning of inflorescences and flowers by the f-box protein DOUBLE TOP and the LEAFY homolog ABERRANT LEAF AND FLOWER of *Petunia* . Plant Cell 20, 2033–2048. doi: 10.1105/TPC.108.060871 18713949 PMC2553618

[B84] Souer E. van Houwelingen A. Kloos D. Mol J. Koes R. (1996). The no apical meristem gene of *Petunia* is required for pattern formation in embryos and flowers and is expressed at meristem and primordia boundaries. Cell 85, 159–170. doi: 10.1016/S0092-8674(00)81093-4 8612269

[B85] Spelt C. Quattrocchio F. Mol J. N. M. Koes R. (2000). *anthocyanin1* of petunia encodes a basic helix-loop-helix protein that directly activates transcription of structural anthocyanin genes. Plant Cell 12, 1619–1631. doi: 10.1105/TPC.12.9.1619 11006336 PMC149074

[B86] Strazzer P. Spelt C. E. Li S. Bliek M. Federici C. T. Roose M. L.. (2019). Hyperacidification of *Citrus* fruits by a vacuolar proton-pumping p-ATPase complex. Nat. Commun. 10, 1–11. doi: 10.1038/s41467-019-08516-3 30808865 PMC6391481

[B87] Stuurman J. Hoballah M. E. Broger L. Moore J. Basten C. Kuhlemeier C. (2004). Dissection of floral pollination syndromes in *Petunia* . Genetics 168, 1585–1599. doi: 10.1534/GENETICS.104.031138 15579709 PMC1448759

[B88] Swamy M. N. Wu H. Shankar P. (2016). Recent advances in RNAi-based strategies for therapy and prevention of HIV-1/AIDS. Adv. Drug Delivery Rev. 103, 174–186. doi: 10.1016/J.ADDR.2016.03.005 PMC4935623 27013255

[B89] Tobeña-Santamaria R. Bliek M. Ljung K. Sandberg G. Mol J. N. M. Souer E.. (2002). FLOOZY of petunia is a flavin mono-oxygenase-like protein required for the specification of leaf and flower architecture. Genes Dev. 16, 753. doi: 10.1101/GAD.219502 11914280 PMC155361

[B90] Turchetto C. Fagundes N. J. R. Segatto A. L. A. Kuhlemeier C. Solís Neffa V. G. Speranza P. R.. (2014). Diversification in the south American pampas: the genetic and morphological variation of the widespread *Petunia axillaris* complex (Solanaceae). Mol. Ecol. 23, 374–389. doi: 10.1111/MEC.12632 24372681

[B91] Vandenbussche M. Chambrier P. Bento S. R. Morel P. (2016). *Petunia*, your next supermodel? Front. Plant Sci. 7. doi: 10.3389/FPLS.2016.00072 PMC4735711 26870078

[B92] Vandenbussche M. Horstman A. Zethof J. Koes R. Rijpkema A. S. Gerats T. (2009). Differential recruitment of WOX transcription factors for lateral development and organ fusion in *Petunia* and *Arabidopsis* . Plant Cell 21, 2269–2283. doi: 10.1105/TPC.109.065862 19717616 PMC2751957

[B93] Vandenbussche M. Janssen A. Zethof J. van Orsouw N. Peters J. van Eijk M. J. T.. (2008). Generation of a 3D indexed petunia insertion database for reverse genetics. Plant J. 54, 1105–1114. doi: 10.1111/J.1365-313X.2008.03482.X 18346192

[B94] Vandenbussche M. Zethof J. Royaert S. Weterings K. Gerats T. (2004). The duplicated b-class heterodimer model: Whorl-specific effects and complex genetic interactions in *Petunia hybrida* flower development. Plant Cell 16, 741–754. doi: 10.1105/TPC.019166 14973163 PMC385285

[B95] van der Krol A. R. Lenting P. E. Veenstra J. van der Meer I. M. Koes R. E. Gerats A. G. M.. (1988). An anti-sense chalcone synthase gene in transgenic plants inhibits flower pigmentation. Nature 333, 866–869. doi: 10.1038/333866a0

[B96] van Houwelingen A. Souer E. Spelt K. Kloos D. Mol J. Koes R. (1998). Analysis of flower pigmentation mutants generated by random transposon mutagenesis in *Petunia hybrida* . Plant J. 13, 39–50. doi: 10.1046/J.1365-313X.1998.00005.X 9680963

[B97] Venail J. Dell’Olivo A. Kuhlemeier C. (2010). Speciation genes in the genus *Petunia* . Philos. Trans. R. Soc. Lond. B. Biol. Sci. 365, 461–468. doi: 10.1098/RSTB.2009.0242 20047872 PMC2838266

[B98] Verhoef N. Yokota T. Shibata K. de Boer G. J. Gerats T. Vandenbussche M.. (2013). Brassinosteroid biosynthesis and signalling in *Petunia hybrida* . J. Exp. Bot. 64, 2435–2448. doi: 10.1093/JXB/ERT102 23599276 PMC3654430

[B99] Verweij W. Spelt C. E. Bliek M. de Vries M. Wit N. Faraco M.. (2016). Functionally similar WRKY proteins regulate vacuolar acidification in *Petunia* and hair development in *Arabidopsis* . Plant Cell 28, 786–803. doi: 10.1105/TPC.15.00608 26977085 PMC4826004

[B100] Verweij W. Spelt C. di Sansebastiano G. Vermeer J. Reale L. Ferranti F.. (2008). An h+ p-ATPase on the tonoplast determines vacuolar pH and flower colour. Nat. Cell Biol. 10, 1456–1462. doi: 10.1038/NCB1805 18997787

[B101] Wang M. Vannozzi A. Wang G. Liang Y. H. Tornielli G. B. Zenoni S.. (2014). Genome and transcriptome analysis of the grapevine (*Vitis vinifera* l.) WRKY gene family. Hortic. Res. 1, 1–16. doi: 10.1038/HORTRES.2014.16 26504535 PMC4596322

[B102] Wheeler L. C. Walker J. F. Ng J. Deanna R. Dunbar-Wallis A. Backes A.. (2022). Transcription factors evolve faster than their structural gene targets in the flavonoid pigment pathway. Mol. Biol. Evol. 39, 1–15. doi: 10.1093/MOLBEV/MSAC044 PMC8911815 35212724

[B103] Wiering H. (1974). Genetics of flower colour in *Petunia hybrida* hort. Genen. en Phaenen. 17, 117–134. doi: 10.3/JQUERY-UI.JS

[B104] Wijsman H. J. W. de Jong J. H. Pedersen T. M. (1983). On the interrelationships of certain species of *Petunia* III. the position of p. linearis and *P. calycina* . Acta Botanica Neerlandica. 32, 323–332. doi: 10.1111/J.1438-8677.1983.TB01717.X

[B105] Yamagishi M. (2020). Isolation and identification of MYB transcription factors (MYB19Long and MYB19Short) involved in raised spot anthocyanin pigmentation in lilies (*Lilium* spp.). J. Plant Physiol. 250, 1–9. doi: 10.1016/J.JPLPH.2020.153164 32460035

[B106] Yarahmadov T. Robinson S. Hanemian M. Pulver V. Kuhlemeier C. (2020). Identification of transcription factors controlling floral morphology in wild *Petunia* species with contrasting pollination syndromes. Plant J. 104, 289–301. doi: 10.1111/TPJ.14962 32780443 PMC7693086

[B107] Zamore P. D. (2006). RNA Interference: big applause for silencing in Stockholm. Cell 127, 1083–1086. doi: 10.1016/J.CELL.2006.12.001 17174883

