# Supplementary material for: The Amsterdam petunia germplasm collection: A tool in plant science
Source: Front Plant Sci. 2023 Mar 21;14:1129724. doi: 10.3389/fpls.2023.1129724 (PMC10070740; doi:10.3389/fpls.2023.1129724)
Supplement: Supplementary file 1 [file DataSheet_1.docx]

>JN065C_Brunfelsia_grandiflora_ODO1

MGIDPVTHEPLNKEANLSNQPNQESDQNKENGHKLEQVVPETTSVTAAATSTELDNNSSSSSSASSSENSSCITEESKMVIDTLGENDPLLSCLLEADAPLMDSPWEFPVSCTTTTEEQKSFDNIISNMTSWDDTLNWLSDCQDFGIDNFGSDNCFNDVEFDIFKTIDDMENKQY

>JG1003B_Calibrachoa_thymifolia_ODO1

MGRQPCCDKLGVKKGPWTAEEDKKLISFILTNGQCCWRAVPKLAGLRRCGKSCRLRWTNYLRPDLKRGLLSDAEEKMVIDLHSRLGNRWSKIAARLPGRTDNEIKNHWNTHIKKKLLKMGIDPVTHEPLQKGASLSDQPNTESDQNIENGHQQVQVVSQGTSVTVAAATSTEIDNNSSFSSSISSSENSSCTTNESKHALDTLSENDPILSCLLEADTPLLDSPWEIPMSSTTTAEEQKSFDNIISNMTSWEDTFNWLSGCQDFGINDFGFDNCFNDVELDIFKTIDIENKHG

>LW012A_Calibrachoa_eglandulata_ODO1

MGRQPCCDKLGVKKGPWTAEEDKKLISFILTNGQCCWRAVPKLAGLRRCGKSCRLRWTNYLRPDLKRGLLSDAEEKMVIDLHSRLGNRWSKIAARLPGRTDNEIKNHWNTHIKKKLLKMGIDPVTHEPLQKGPSLSDQPNTESDQNIENGHQQVQVVSQGTSVTVAAATSTEIDNNSSFSSSTSSSENSSCTTNESKHALDTLSENDPILSCLLEADTPLLDSPWEIPMSSTTTAEEQKSFDNIISNMTSWEDTFNWLSGCQDFGINDFGFDNCFNDVELDIFKTIDIENKHG

>RD382F_Fabiana_denudata_ODO1

GRQPCCDKLGVKKGPWTAEEDKKLISFILTNGQCCWRAVPKLAGLRRCGKSCRLRWTNYLRPDLKRGILSDAEEKLVIDLHSRLGNRWSKIAARLPGRTDNEIKNHWNTHIKKKLLKMGIDPVTHEPLKKEANLRDQPNTESDEDKENGHQQVQVVPQSTNVTAAAATSTDLDNNSSSSSSASSSQNSSCTTNESKLVLDNLSEDDPLLSCLLEADTPLIDSSWEFPMSSTTTAEEQKCFDNIISNMTSWEDTFNWLSGCQDFGINDFGFDNCFNDVELDIFQTIDNVEIKHG

>LW014A_Petunia_altiplana_ODO1

MGRQPCCDKLGVKKGPWTAEEDKKLISFILTNGQCCWRAVPKLAGLRRCGKSCRLRWTNYLRPDLKRGLLSDAEEKLVIDLHSRLGNRWSKIAARLPGRTDNEIKNHWNTHIKKKLLKMGIDPVSHEPLKKEANLSDQPNTESDQNKENGHQQVQVVPQSTNVTAAAATSTEFDNNSSFSSSASSSENSSCTTNESKLVFDNLSENDPLLSCLLEADTPLIDSPWEFPMSSTTTAEEPKSFNSIISNMTSWEDTFNWLSGCQEFGINDFGFDNCFNHIELDIFKTIDNVENRHG

>JN042VOUCH_Petunia_secreta_ODO1

MGRQPCCDKLGVKKGPWTAEEDKKLISFILTNGQCCWRAVPKLAGLRRCGKSCRLRWTNYLRPDLKRGLLSDAEEKLVIDLHSRLGNRWSKIAARLPGRTDNEIKNHWNTHIKKKLLKMGIDPVTHEPLKKEANLSDQPTTESDQNKENGHQQVQVVPQSTNVIAAAATSTEFDNNSSFSSSASSSENSSCTTNESKLVFDNLSENDPLLSCLLEADTPLIDSPWEFPMSSTTTVEEPKSFDSIISNMTSWEDTFNWLSGYQEFGINDFGFDNCFNHIELDIFKTIDNVENRHG

>RD387B_Fabiana_bryoides_ODO1

MGRQPCCDKLGVKKGPWTAEEDKKLISFILTNGQCCWRAVPKLAGLRRCGKSCRLRWTNYLRPDLKRGILSDAEEKLVIDLHSRLGNRWSKIAARLPGRTDNEIKNHWNTHIKKKLLKMGIDPVTHEPLKKEANLRDQPNTESDEDKENGHQQVQVVPQSTNVTAAAATSTDLDNNSSSSSSASSSQNSSCTTNESKLVLDNLSENDPLLSCLLEADTPLIDSSWEFPLSSTTTAEEQKSFDNIISNMTSWEDTFNWLSGCQDFGINDFGFDNCFNDVELDIFKTIDNVEIKHG

>LW022A_Petunia_interior_ODO1

MGRQPCCDKLGVKKGPWTAEEDKKLISFILTNGQCCWRAVPKLAGLRRCGKSCRLRWTNYLRPDLKRGLLSDAEEKLVIDLHSRLGNRWSKIAARLPGRTDNEIKNHWNTHIKKKLLKMGIDPVTHEPLKKEANLSDQPNTESDQNKENGHQQVQVVPQSTNVTAAAATSTEFDNNSSFSSSASSSENSSCTTNESKFVFDNLSENDPLLSCLLEADTPLIDSPWEFPMSSTTTAEEPKSFDSIISNMTSWEDTFNWLSGCQEFGINDFGFDNCFNHVELDIFKTIDNVENRHG

>RD420B_Leptoglossis_acutiloba_ODO1

KKLISFILTNGQCCWRAVPKLAGLRRCGKSCRLRWTNYLRPDLKRGLLSDEEEKLVIDLHARLGNRWSKIAAKLPGRTDNEIKNHWNTHIKKKLLKMGIDPVTHEPLNKDVNLSDQPKPESDKNKENGDQQVRVVPESTSVTAAATSTELDNNSSFSSSASSSENSSCTTNETKLVVDTLISENDPLLSCLLEADYAPLTDSPWEIPVSHTEQQKCIDNIIDNMTSWDDTLNWLSDSQDFGIDDFGFDNCFNDIVEEFNIFKTIDHDVENKH

>SS678A_Bouchetia_erecta_ODO1

MGRQPCCDKLGVKKGPWTAEEDKKLINFILTNGQCCWRAVPKLAGLRRCGKSCRLRWTNYLRPDLKRGLLSDAEEKLVIDLHARLGNRWSKIAARLPGRTDNEIKNHWNTHIKKKLLKMGIDPVTHEPLSNKEANLSDQRNSESDQVKENGHQQVQVVPESTSVTAPELDSNSSSSSSPSSSENSSCTTNESKLVLDPLDESDPLLSCLLEADHAPLIDSPWEIPMSRTEEQKSIDNIISNMTSWDDTLNWLSDCQDFGIDDFGFDNCFNDIVEFDIFKTIDHDMVENKH

>JG1090A_Calibrachoa_pubescens_ODO1

MGIDPVTHEPLQKGASLSDQPNTESDQNIENGHQQVQVVSQGTSVTVAAATSTEIDNNSSFSSSISSSENSSCTTNESKHALDTLSENDPILSCLLEADTPLLDSPWEIPMSSTTTAEEQKSFDNIISNMTSWEDTFNWLSGCQDFGINDFGFDNCFNDVELDIFKTIDIENKHG

>LW011VOUCH_Petunia_reitzii_ODO1

MGRQPCCDKLGVKKGPWTAEEDKKLISFILTNGQCCWRAVPKLAGLRRCGKSCRLRWTNYLRPDLKRGLLSDAEEKLVIDLHSRLGNRWSKIAARLPGRTDNEIKNHWNTHIKKKLLKMGIDPVTHEPLNKEANLSDQPNTESDQNKQNGHQQVQVVPQSTNVTAAAATSTEFDNNSSFSSSASSSENSSCTTNESKLVFDNLSENDPLLSCLLEADTPLIDSLWEFPMSSTTTAEEPKSFDNIISNMTSWEDTFNWLSGCQEFGINDFGFDNCFNHVELDIFKTIDNVENRHG

>JN041A_Petunia_integrifolia_ODO1

MGRQPCCDKLGVKKGPWTAEEDKKLISFILTNGQCCWRAVPKLAGLRRCGKSCRLRWTNYLRPDLKRGLLSDAEEKLVIDLHSRLGNRWSKIAARLPGRTDNEIKNHWNTHIKKKLLKMGIDPVTHEPLKKEANLSDQPNTESDQNKENGHQQVQVVPQSTNVTAAAATSTEFDNNSSFSSSASSSENSSCTTNESKLVFDNLSENDPLLSCLLEADTPLIDSPWEFPMSSTTTAEEPKSFDSIISNMTSWEDTFNWLSGCQDFGINDFGFDNCFNHIELDIFKTIDNVENRHG

>JG1007VOUCH_Calibrachoa_longistyla_ODO1

ENSSCTTNESKHALDTLSENDPILSCLLEADTPLLDSPWEIPMSSTTTAEEQKSFDNIISNMTSWEDTFNWLSGCQDFGINDFGFDNCFNDVELDIFKTI

>LW005A_Fabiana_densa_ODO1

MGRQPCCDKLGVKKGPWTAEEDKKLISFILTNGQCCWRAVPKLAGLRRCGKSCRLRWTNYLRPDLKRGILSDAEEKLVIDLHSRLGNRWSKIAARLPGRTDNEIKNHWNTHIKKKLLKMGIDPVTHEPLKKEANLRDQPNTESDEDKENGHQQVQVVPQSTNVTAAAATSTDLDNNSSSSSSASSSQNSSCTTNESKLVLDNLSENDPLLSCLLEADTPLIDSSWEFPMSSTTTAEEQKSFDNIISNMTSWEDTFNWLSGCQDFGINDFGFDNCFNDVELDIFKTIDNVEIKHG

>LW009A_Calibrachoa_sendtneriana_ODO1

VKKGPWTAEEDKKLISFILTNGQCCWRAVPKLAGLRRCGKSCRLRWTNYLRPDLKRGLLSDAEEKMVIDLHSRLGNRWSKIAARLPGRTDNEIKNHWNTHIKKKLLKMGIDPVTHEPLQKGASLSDQPNTESDQNIENGHQQVQVVSQGTSVTVAAATSTEIDNNSSFSSSISSSENSSCTTNESKHALDTLSENDPILSCLLEADTPLLDSPWEIPMSSTTTAEEQKSFDNIISNMTSWEDTFNWLSGCQDFGINDFGFDNCFNDVELDIFKTIDIENKHG

>SS605A_Petunia_axillaris_ODO1

KEANLSDQPTTESDQNKENGHQQVQVVPQSTNVTAAAATSTEFDINSSFSSSASSSQNSSCTTNESKLVFDNLSENDPLLSCLLEADTPLIDSPWEFPMSSTTTVEEPKSFDSIISNMTSWEDTFNWLSGYQEFGINDFGFDNCFNHVELDIFKTIDNVENRHG

>JN048VOUCH_Calibrachoa_sellowiana_ODO1

MGRQPCCDKLGVKKGPWTAEEDKKLISFILTNGQCCWRAVPKLAGLRRCGKSCRLRWTNYLRPDLKRGLLSDAEEKMVIDLHSRLGNRWSKIAARLPGRTDNEIKNHWNTHIKKKLLKMGIDPVTHEPLQKGASLSDQPNTESDQNIENGHQQVQVVSQGTSVTVAAATSTEIDNNSSFSSSISSSENSSCTTNESKHTLDTLSENDPILSCLLEADTPLLDSPWEIPMSSTTTAEEQKSFDNIISNMTSWEDTFNWLSGCQDFGINDFGFDNCFNDVELDIFKTIDIENKHG

>LW023A_Petunia_correntina_ODO1

MGRQPCCDKLGVKKGPWTADEDKKLISFILTNGQCCWRAVPKLAGLRRCGKSCRLRWTNYLRPDLKRGLLSDAEEKLVIDLHSRLGNRWSKIAARLPGRTDNEIKNHWNTHIKKKLLKMGIDPVTHEPLKKEANLSDQPTTESDQNKENGHQQVQVVPQSTNVTVAAATSTEFDNNSSFSSSASSSENSSCTTNESKLVFDNLSENDPLLSCLLEADTPLIDSPWQFPMSSTITAEEPKSFDNIISNMTSWEDTFNWLSGCQEFGINDFGFDNCFNHIELDIFKTIDNVENRHG

>JN060VOUCH_Calibrachoa_irigangiana_ODO1

HEPLQKGASLSDQPNTESDQNIENGHQQVQVVSQGTSVTVAAATSTEIDNNSSFSSSISSSENSSCTTNESKHTLDTLSENDPILSCLLEADTPLLDSPWEIPTSSTTTAEEQKSFDNIISNMTSWEDTFNWLSGCQDFGINDFGFGNCFNDVELDIFKTIDIENKHG

>SS622B_Nierembergia_browallioides_ODO1

GNRWSKIAARLPGRTDNEIKNHWNTHIKKKLLKMGIDPVTHEPLNSKEANLSDQQNSESDQNKENGHQQVQVVPESTNVTAPEFDNKSSSSSSASSSENSSSTTNESKLVLDPLSESDPLLSCLLEADHAPLIDSSWEIPVSSTDEQQKSFD

>SS668C_Nierembergia_graveolens_ODO1

ENLSDQLNSESDQNKENGHQQVQVVPESTNVIAPAELENNSSSSSSASSSENSSCTTNESKLVLDPLSENDPLLSCLLEADHAPLIDSSWEIPVSSTDEQQKSFNNIISNMTSWDDTLNWLSDGQDFGIDDFGIDNCFNDIIEFDIFKTLDHEIVEINKH

>JN049VOUCH_Petunia_bonjardensis_ODO1

MGRQPCCDKLGVKKGPWTAEEDKKLISFILTNGQCCWRAVPKLAGLRRCGKSCRLRWTNYLRPDLKRGLLSDAEEKLVIDLHSRLGNRWSKIAARLPGRTDNEIKNHWNTHIKKKLLKMGIDPVTHEPLKKEANISDQPNTESDQNKENGHQQVQVVPQSTDVTAAAATSTEFDNNSSFSSSASSSENSSCTTNESKLVFDNLSENDPLLSCLLEADTPLIDSPWEFPMSSTTTAEEPKSFDSIISNMTSWEDTFNWLSGCQEFGINDFGFDNCFNHVELDIFKTIDNVENRQDNCPI

>MSBG-1985-420_Brunfelsia_pauciflora_ODO1

VKKGPWTAEEDKKLINFILTNGQCCWRAVPKLAGLRRCGKSCRLRWTNYLRPDLKRGLLSDAEEKLVIDLHARLGNRWSKIAARLPGRTDNEIKNHWNTHIKKKLLKMGIDPVTHEPLNKEANRSNQPNQES

>JN078A_Brunfelsia_jamaicensis_ODO1

MGIDPVTHEPLKNLSDQPNQESDQNKENGHEQLQVVPESTSVTAAATSTELDNNSSSSSSASSSENSSCITEESKLVIDTLGENDPLLSSLLEADAPLIDSPWEFPVSCTTTTEEQKGLITLLVT

>SS618A_Nierembergia_tucumanensis_ODO1

KKLLKMGIDPVTHEPLNNKEANLSDQPNSESDQNKENGHQLVQVVPESTNVTAPELDNNSSSSSSASSSENSSSTTNESKLVLDPLSESDPLLSCLLEADHAPLIDSSWEIPVSSTDEQQKSFDNIISNMTSWDDTLNWLSDGQDFGIDDFGIDNCFNDIVEFDIFKTLDHEIVEINKH

>LW019A_Nierembergia_rivularis_ODO1

MGRQPCCDKLGVKKGPWTAEEDKKLINFILTNGQCCWRAVPKLAGLRRCGKSCRLRWTNYLRPDLKRGLLSDAEEKLVIDLHARLGNRWSKIAARLPGRTDNEIKNHWNTHIKKKLLKMGIDPVTHEPLNNKVTNLSDQPNSESDQNKENGHQQVEVVPESTNVTAPELDNNSSSSSSASSSENSSSTTNESKLVLDPLSESDPLLSCLLEADHAPLIDSSWEIPVSCTEQQKSLDNIISNMTSWDDTLNWLSDGQDFGIDDFGIDDYCFNDIVEFDIFKTIDHEIVEINKH

>SS620A_Fabiana_patagonica_ODO1

MGRQPCCDKLGVKKGPWTAEEDKKLISFILTNGQCCWRAVPKLAGLRRCGKSCRLRWTNYLRPDLKRGILSDAEEKLVIDLHSRLGNRWSKIAARLPGRTDNEIKNHWNTHIKKKLLKMGIDPVTHEPLKKEANLRDQPNTESDEDKENGHQQVQVVPQSTNVTAASATSTDLDNNSSSSSSASSSQNSSCTTNESKLVLDNLSENDPLLSCLLEADTPLIDSSWEFPMSSTTTTAEEQKSFDNIISNMTSWEDTFNWLSGCQDFGINDFGFDNCFNDVELDIFKTIDNVEIKHG

>LW017A_Petunia_scheideana_ODO1

CWRAVPKLAGLRRCGKSCRLRWTNYLRPDLKRGLLSDSEEKLVIDLHSRLGNRWSKIAARLPGRTDNEIKNHWNTHIKKKLLKMGIDPVTHEPLKKEANLSDQPNTESDQNKENGHQQVQVVPQSTNVTAAASTSTEFDNNSSFSSSASSSENSSCTTNESKLVFDNLSENDPLLSCLLEADTPLIDSPWEFPMSSTTTTAEEPKSFDNIISNMTSWEDTFNWLSGCQDFGINDFGFDNCFNHVELDIFKTIDNVENRHG

>SS663D_Calibrachoa_missionica_ODO1

MGRQPCCDKLGVKKGPWTAEEDKKLISFILTNGQCCWRAVPKLAGLRRCGKSCRLRWTNYLRPDLKRGLLSDAEEKMVIDLHSRLGNRWSKIAARLPGRTDNEIKNHWNTHIKKKLLKMGIDPVTHEPLQKGASLSDQPNTESDQNIENGHQQVQVVSQGTSVTVAAATSTEIDNNSSFSSSISSSENSSCITNESKHALDTLSENDPILSCLLEADTPLLDSPWEIPMSSTTTAEEQKSFDNIISNMTSWEDTFNWLSGCQDFGINDFGFDNCFNDVELDIFKTIDIENKHG

>SS679A_Fabiana_imbricata_2019_ODO1

MGRQPCCDKLGVKKGPWTAEEDKKLISFILTNGQCCWRAVPKLAGLRRCGKSCRLRWTNYLRPDLKRGILSDAEEKLVIDLHSRLGNRWSKIAARLPGRTDNEIKNHWNTHIKKKLLKMGIDPVTHEPLKKEANLGDQPNTESDQDKENGHQQVQVVPQSTNVTAAAATSTDLDNNSSSSSSASSSQNSSCTTNESKLVLDNLSENDPLLSCLLEAETPLIDSSWEFPMSSTTTTAEEQKSFDNIISNMTSWEDTFNWLSGCQDFGINDFGFDNCFNDVELDIFKTIDNVEI

>LW010A_Calibrachoa_linoides_ODO1

MGRQPCCDKLGVKKGPWTAEEDKKLISFILTNGQCCWRAVPKLAGLRRCGKSCRLRWTNYLRPDLKRGLLSDAEEKMVIDLHSRLGNRWSKIAARLPGRTDNEIKNHWNTHIKKKLLKMGIDPVTHEPLQKGASLSDQPNTESDQNIENGHQQVQVVSQGTSVTVAAATSTEIDNNSSFSSSISSSENSSCTTNESKHTLDTLSENDPILSCLLEADTPLLDSPWEIPMSSTTTAEEQKSFDNIISNMTSWEDTFNWLSGCQDFGINDFGFDNCFNDVELDIFKTIDIENKHG

>LW007A_Calibrachoa_heterophylla_ODO1

FILTNGQCCWRAVPKLAGLRRCGKSCRLRWTNYLRPDLKRGLLSDAEEKMVIDLHSRLGNRWSKIAARLPGRTDNEIKNHWNTHIKKKLLKMGIDPVTHEPLQKGASLSDQPNTESDQNIENGHQQVQVVSEGTSVTVAAATSTEIDNNSSFSSSISSSENSSCTTNESKHTLDTLSENDPILSCLLEADTPLLDSPWEIPMSSTTTAEEQKSFDNIISNMTSWEDTFNWLSGCQDFGINDFGFDNCFNDVELDIFKTIDIENKHG

>JN001A_Petunia_exserta_ODO1

LKMGIDPVTHEPLKKEANLSDQPTTESDQNKENGHQQVQVVPQSTNVTAAAATSTEFDNNSSFSSSASSSENSSCTTNESKLVFDNLSENDPLLSCLLEADTPLIDSPWEFPMSSTTTVEEPKSFDSIISNMTSWEDTFNWLSGYQEFGINDFGFDNCFNHVELDIFKTIDNVENRHG

>JG1004A_Calibrachoa_caesia_ODO1

ENGHQQVQVVSQGTSVTVAAATSTEIDNNSSFSSSTSSSENSSCTTNESKHALDTLSENDPILSCLLEADTPLLDSPWEIPMSSTTTAEEQKSFDNIISNMTSWEDTFNWLSGYQDFGINDFGFDNCFNDVELDIFKTIDIENKHG

>JN055VOUCH_Calibrachoa_elegans_ODO1

MGRQPCCDKLGVKKGPWTAEEDKKLISFILTNGQCCWRAVPKLAGLRRCGKSCRLRWTNYLRPDLKRGLLSDAEEKMVIDLHSRLGNRWSKIAARLPGRTDNEIKNHWNTHIKKKLLKMGIDPVTHEPLQKGASLSDQPNTESDQNIENGHQQVQVVSQGTSVTVAAATSTEIDNNSSFSSSTSSSENSSCTTNESKNSSCTTNESKHALDNLSENDPILSCLLEADTPLLDSPWEIPMSSTTTAEEQKSFDNIISNMTSWEDTFNWLSGCQDFGINDFGFDNCFNDVELDIFKTIDIENKHG

>SS627B_Nierembergia_pulchella_ODO1

THIKKKLLKMGIDPVTHEPLNNKEANLSDQPNSESDQNKENGHQLVQVVPESTNVTAPELDNNSSSSSSASSSENSSSTTNESKLVLDPLSESDPLLSCLLEADHAPLIDSSWEIPVSSTDEQQKSFDNIISNMTSWDDTLNWLSDGQDFGIDDFGIDNCFNDIVEFDIFKTLDHEIVEINKH

>SS626A_Fabiana_friesii_ODO1

MGRQPCCDKLGVKKGPWTTEEDKKLINFLIKNIGQCCWRSVPKLAGLRRCGKSCRLRWTNYLRPDLKRGLLTEAEEKLVIELHSCLGNRWSKIAARLPGRTDNEIKNHWNTHIKKKLLKMGIDPVTHEPLKKEANLRDQPNTESDEDKENGHQQVQVVPQSTNVTAAAATSTDLDNNSSSSSSASSSQNSSCTTNESKLVLDNLSENDPLLSCLLEADTPLIDSSWEFPMSSTTTAEEQKSFDNIISNMTSWEDTFNWLSGCQDFGINDFGFDNCFNDVELDIFKTIDNVEIKHG

>LW026A_Fabiana_peckii_ODO1

KKGPWTAEEDKKLISFILTNGQCCWRAVPKLAGLRRCGKSCRLRWTNYLRPDLKRGILSDAEEKLVIDLHSRLGNRWSKIAARLPGRTDNEIKNHWNTHIKKKLLKMGIDPVTHEPLKKEANLRDQPNTESDEDKENGHQQVQVVPQSTNVTAAAATSIDLDNNSSSSSSASSSQNSSCTTNESKLVLDNLSENDPLLSCLLEADTPLIDSSWEFPMSSTTTAEEQKCFDNIISNMTSWEDTFNWLSGCQDSSINDFGFDNCFNDVELDIFQTIDNVEIKHG

>SS624C_Fabiana_punensis_ODO1

MGIDPVTHEPLKKEANLRDQPNTESDEDKENGHQQVQVVPQSTNVTAAAATSTDLDNNSSSSSSASSSQNSSCTTNESKLVLDNLSENDPLLSCLLEADTPLIDSSWEFPMSSTITAEEQKCFDNIISNMTSWEDTFNWLSGCQDSGINDFGFDKCFNDVELDIFQTIDNVEIKHG

>LW020A_Petunia_inflata_ODO1

MGRQPCCDKLGVKKGPWTAEEDKKLISFILTNGQCCWRAVPKLAGLRRCGKSCRLRWTNYLRPDLKRGLLSDAEEKLVIDLHSRLGNRWSKIAARLPGRTDNEIKNHWNTHIKKKLLKMGIDPVTHEPLKKEANLSDQPNTESDQNKENGHQQVQVVPQSTNVTAAAATSTEFDNNSSFSSSASSSENSSCTTNESKLVFDNLSENDPLLSCLLEADTPLIDSPWEFPMSSITTAEEPKSFDNIISNMTSWEDTFNWLSGCQEFGINDFGFDNCFNHVELDIFKTIDNVENRHG

>Soltu.DM.08G025560_1_ODO1

MGRQPCCDKLGVKKGPWTAEEDKKLINFILTNGQCCWRAVPKLAGLRRCGKSCRLRWTNYLRPDLKRGLLSEAEEKLVIDLHARLGNRWSKIAARLPGRTDNEIKNHWNTHIKKKLLKMGIDPVTHEPLIKAENPADKNKNNDHQLVVVVPESTSVTAAAISSELENSSTSSSSASSSENSCNESKLVLDTFNENDPLLNSLLESDVTPLINSSWELPIKSFDNIINNSNNHMTSSWDDESFNWLLDCQDFGIHDFGFDNCFNNDVELEVFNTKFDMENKK

>JN056VOUCH_Brunfelsia_brasilensis_MYBFL

MVRAPCCEKVGLKRGRWTAEEDELLFKYIQANGEGSWRSLPKNADTTTTRECGLLHGFVYKGLSRCGKSCRLRWTNYLRPNLKRGKFTTEEDETIVKLQHSLGNRWALIASYLPGRTDNEIKNYWNSHLRRKIYTFSENNNTSIKTTVELAKKITNIADGINSESLRKRGRVSRSKAKKYNNNSTTTTFVSTLKTKSSCVGRSGRATRPNSSENFTINREFLALPLLEETGLAIQQHECLVESAIGDPRNEEAEDNNQKDINATPRVQEECNGVLSLGEQRRKILDEHNNIEGPHYKNGSDETRLVQQPNYFLHDFDNQVSLSGALEKNEVGENWWSTMISDHFLEDELWVDQCNSLDLEFGSIEECTTCCDDDMLLWLWDDN*

>RD382F_Fabiana_denudata_ MYBFL

MVRAHCCEKVGLKRGRWTAEEDDLLLKYIQANGEGLWKSLPKHAGLLRCGKSCRLRWTNYLRPNLKRGKFTSEEDETIVKLQCSLGNRWSLIASCLPGRTDNEIKNYWNSHLRRRIYTFRMKKKPIKTTAEVPKKTIVADGINCEFLRKRGRVSRSKAKKYNSNTTTTATAYISSLKPKSSCVGAGSRATWSDGDSIEFHSSNFFTIDTGNKFHTHKAYACCIFIFYP*

>Ventura_Fabiana_viscosa_ MYBFL

GKFTSEEDETIVKLQCSLGNRWSLIASCLPGRTDNEIKNYWNSHLRRRIYTYRMKNKTIKTTAEVPKNTIVADGINCEFLRKRGRVSRSKAKKYNSNTTTTATAYISTLKPKTSCVGAGSRAIWSDGDSIEFHLSENFTIDTETLALLEGKGQAIQQHDEHDAGSAIGKPRNEETEGK

>JN042VOUCH_Petunia_secreta_ MYBFL

EISFPHPAPPKAHSLYLIAVCHYVICTNVSKDSLAISLTEMVRAPCCEKVGIKRGRWTAEEDELLLKYIQANGEGSWRSLPKNAGLLRCGKSCRLRWTNYLRPNLKRGKFTSEEDETIFKLQCSLGNRWSLMASYLPGRTDNEIKNYWNSHLRRRIYTFGMKKKPIKTAAEMPNKTIVADGLNCESLKKRGRVSRSKAKKYNNNTTTTTTAYISTLKPKSSGVGAGGGAICSEGDSIVDTGIGIDIQQHDEDQAGSAIGKPRNEETEGTNQKHINATAEKQEVRNGILSFEEQGQQVLDEHILIGPHEKNVGDETVHLQQPNYCLHDFGNQVSLSGVLEVDEESHENWWSTMNSDNFLEDELWVDQCSSLDLEFGSIEECDDMLLGIFYY*

>JN076A_Bouchetia_anomala_ MYBFL

MVKAPCCEKVDLKKGRWTAEEDELLLNYIQTNGEGFWRSLPKNAGLLRCGKSCRLRWINYLRPNLKRGKFTAEEDETIVKLQHSLGNRWSLIATYLPGRTDNEIKNYWNCHLRRILYTFMINNKSIKKTADPVPQNLLVADDTNCKSLRKQGRVSRSKAKKYSNDTTTTYVSKLEPKSLRLNDRGEDTLSEKDSIESHFF*

>RD387B_Fabiana_bryoides_ MYBFL

MVRAPCCEKVGLKRGRWTAEEDDLLLKYIQANGEGLWKSLPKNAGLLRCGKSCRLRWTNYLRPNLKRGKFISEEDETIVKLQCSLGNRWSLIASCLPGRTDNEIKNYWNSHLRRRIYTFRMKKKPIKTTAEVPKKTIVADGINCEFLRKRGRVSRSKAKKYNGNTTTTATAYISSLKPKSSCVGAGSRATWSDGDSIEFHLSENFTVDTANKFHTYKAYACCIFIFYP*

>SS689A_Brunfelsia_nitida_ MYBFL

MCYRWALIASYLPGRTDNEIKNYWNSHLRRKIYTFRVNNSRSIKTTVELPKKITNVADGINSESLRKSGRVSRSKAKKYNNNSTTTTFVSTLKPKSSCVGRSGRATRPNSSENFTIDKEFLALQLLEETGLAIQQHECLLESAIGDPRNEEAEDNNQKDINATARVQEVCNGVWSLEEQRRKVLDEHNNTKGPHDKNGSDETTLVQQPNYCLHDFDNQVSLSGVLEMNEVGEN*

>JG1090A_Calibrachoa_pubescens_ MYBFL

MVRAPCCEKAGLKRGRWTAEEDELLLKYIQANGEGSWRSLPKSAGLLRCGKSCRLRWTNYLRPNLKRGKFTSEEDETIVKLQCSLGNRWSLIASYLPGRTDNDIKNYWNSQLRRKIYTFRMKKKPIKTTAEVPKMTIVADGINCESLRKCGRVSRSKAKKYNHNTTTTATAYISSLKPKSSCVNVGAHSGATWSEGDSIEFDLSEIFTIDTAYLATLEGIGLDMQQHDEHYAGSAIGKPRNVEREGNNEKHINATPEEEEVCHGMWSFEETEGKNQKQINATPEKQLEVCNGMWSFEETEGKNQKQINATPEEEEVCHGMWSFEETEGKNQKQINATPEKQLEVCNGMWFFVEQGQQVLDEQIPNGPHETNAGDETVYLQQPNYCLHDFDNQVTLSGVLEINEESHENWWSAMTSDNCLEDELWVDQCSSMDGEFGSNEEFDDMLLWLWDDI*

>JN041A_Petunia_integrifolia_ MYBFL

PKNAGLLRCGKSCRLRWTNYLRPNLKRGKFTSEEDETIVKLQCSLGNRWSLMASYLSGRTDNEIKNYWNSHLRRRIYTFRMKTKPIKTVAEVPNKTIVADGLNCEYLKKHGRVSRSKAKKYNNNITTTTTAYISTFKPKSSCVGAGGGATCSEGDSIVDTGVGLDMQQHDEHHARSAIHKPRNEETEGKNQKHINATPEKQEVCNGMWSIEEHEGQQVLDEHIPIGPHEKNISDETVHLQQTNYCLNDFGNQVSLSGVLEVDDESHENWWSTMNSDNFLEDELWVDQCSSLDLEFGSIEECDHMLLSLWDDN*

>LW005A_Fabiana_densa_ MYBFL

MVTAPCCEKVGLKRGRWTAEEDDLLLKYIQANGEGLWKSLPKNAGLLRCGKSCRLRWTNYLRPNLRRGKFTSEEDETIVKLQCSLGNRWSLIASCLPGRTDNEIKNYWNSHLRRRIYNFRMKKKPIKTTAEVPKKTIVADAINCEFLRKRGRVSRSKAKKIQ*

>SS605A_Petunia_axillaris_ MYBFL

EISFPHPAPPKAHSLYLIAVCHYVICTNVSKDSLAISLTEMVRAPCCEKVGIKRGRWTAEEDELLLKYIQANGEGSWRSLPKNAGLLRCGKSCRLRWTNYLRPNLKRGKFTSEEDETIFRLQCSLGNRWSLMASYLPGRTDNEIKNYWNSHLRRRIYTFGMKKKPIKTAAEMPNKTIVADGLNCESLKKRGRVSRSKAKKYNNNTTTTTTAYISTLKPKSSGVGAGGGAICSEGDSIVDTGKKFHTHKSIFLLISNSTLNNSLKNDITCLRFIHHSLKLQDSSFLLHSSGTNENI*

>JN048VOUCH_Calibrachoa_sellowiana_ MYBFL

LRPNLKRGKFTSEEDETIVKLQCSLGNRWSLIASYLPGRTDNEIKNYWNSHLRRRIYTFRMKKKPIKTTAEVPKMTIVADGINCESLRKCGRVSRSKAKKYNHNTTTTATAYISSLKPKSSCVNVEAHSGATWSEGDSIEFDLSEIFTIDTAYLATLEGIGLDMQQHDEHYAGSAIGKPRNVEREGNNEKHINATPEEEEVCHGMWSFEETEGKNQKKFNATPDKQLEVCNGMWSFEETEGKNQKQINATPEKQLEVCNGMWFFEEQGQQVLDEQIPNGPHETNAGDETVYLQQPNYCLHDFDNQVTLSGVLEINEESHENWWSAMTSDNFLEDELWVDQCSSMDVEFGSNEEFDDMLLWLW

>JN060VOUCH_Calibrachoa_irigangiana_ MYBFL

MNYFSSIFKLMEKARGGLLRCGKSCRLRWTNYLRPNLKRGKFTSEEDETIVKLQCSLGNRWSLIASYLPGRTDNDIKNYWNSHLRRRIYTFRMKKKPIKTTAEVPKMTIVADGINCESLRKCGRVSRSKAKKYNHNTTTTATAYISSLKPKSSWVNVGAHSGATWSEGDSIEFDLSEIFTIDTAYLATLEGIGLDMQQHDEHYAGSAIGKPRNVEREGNNEKHINATPEEEEVCHGMWSFEETEGKNQKQINATPEKQLEVCNGMRSFEETEGKNQKQINATPEKQLEVCNGMWSFEETEGKNQKQINATPEKKLEVCNGMWSFEETEGKNQKQINATPEKQLEVCNGMWSFEETEGKNQKQINATPEKQLEVCNGMWFFEEQVLDEQIPNGPHETNAGDETVYNLQQPNYSLHDFDNQVTLSGVLEINEESHENWWSAMTSDNCLEDELWVDQCSSMDVEFGSNEEFDDMLLWLWDDI*

>SS609C_Leptoglossis_linarifolia_ MYBFL

IRYKKMVKVPCGEKVRLKKGRWTTEEDELLLNYIQSHGEGSWRSLPKNAGLLRCGKSCRLRWLNYLRPNLRRGKFTQEEDETIVKLQHSLGNRWSLIASYLPGRTDNEIKNYWNSHLRRILYTFKMNNKSTKKTTDQIPNVVDGINCEPLRKQGRVSRSKAKKYNNNTTTATTSISNEKSKSLRPEDTGKGTWSKGNLTDSQLAENFTVDKECLVLPLLEENSIDESRNEDAADENENSIIINATPEEQDEHILTGPHDQENQSVAMQQLHEFDHFLEEELRVYEFSKSLDSEFGSIDQEITAYFDDSLWQWNDN*

>JN078A_Brunfelsia_jamaicensis_ MYBFL

MCYRWALIASYLPGRTDNEIKNFWNSHLRRKIYTFRVNNSRSIKTTVELPKTITNVADGINSESLRKRGRVSRSKAKKYNNNSTTTTFVSTLKPKSSCVGRSGRATRPNSSENFTIDKEFLALQLLEETGLAIQQHECLLESAIGDPRNEEAEDNNQKDINATARVQEVCNGEWSFEEQRRKVLDEHNNTKGPHDKNGSDETTLVQQHNYCLHDFDNQVSLSGVLEMNEVGEN*

>SS618A_Nierembergia_tucumanensis_MYBFL

MGRAPCCEKVGLKRGRWSSQEDELLVKYMQANGEGSWRTLPKNAGLLRCGKSCRLRWINYLKPNLKRGKFTSQEDETIVKLQYSLGNRWSLISTYLPGRTDNEIKNYWNSHLRRRMCTFMLKNKHIEKTTLELPKRTIVAGKSLRKQGRVSLSKAKKYNNNGNTTNTYVSNLKIKSSCLGADSGGGATWSGGDYSVESHLSKKFTVDTELLTSPQLEEETRLPMQQNHATPENQEVCNGMWSFDKEGGHEQILLDHDDQHLQIIGPLEKNEGDVSVLLEKPNYCLNEFEEGKNWWSTMNISDNNFLEDELWVQDQCNFSLDMDFGSIEESTTYCDDDDDD*

>LW019A_Nierembergia_rivularis_ MYBFL

MGRAPCCEKLGLKRGRWSSEEDELLVKYIQANGEGSWRSLPKNAGLLRCGKSCRLRWINYLKPNLKRGKFTSQEDETIVKLQYSLGNRWSLISTYLPGRTDNEIKNYWNSHLRRRMYTFMLKNKHIEKTTLEFPKRTIVAAKSLRKQGRVSRSKAKKYNNNSNITTTYVSTLKIKSSCLGVGGGGSGGATWSGRDSLGSHLSKKFTVDTEFLASPQHEEETRLAMQQNHATSENKKVCNGMWSFDQEKGYEQILLDHDDDQHIQIIGPLEKNEGDVSVLLEKPNYCLNEFEEGKNWWSTMNISGNYLDDDLWIQDQYNFMS*

>SS663D_Calibrachoa_missionica_ MYBFL

MVRAPCCEKAGLKRGRWTAEEDELLLKYIQANGEGSWRSLPKSAGLLRCGKSCRLRWTNYLRPNLKRGKFTSEEDETIVKLQCSLGNRWSLIASYLPGRTDNEIKNYWNSHLRRRIYTFRMKKKPIKTTAEVPKMTIVADGINCESLRKCGRVSRSKAKKYNHNTTTTATAYISSLKPKSSCVNVGAHSGATWSEGDSIEFDLSEIFTIDTAYLATLEGIGLDMQQHDEHYAGSAIGKPRNVEREGNNEKHINATPEEEEVCHGMWSFEETEGKNQKQINATPEKQLEVCNGMWSFEETEGKNQKQINATPEKQLEVCNGMWFFEEQGQQVLDEQIPNGPHETNAGDETVYLQQPNYCLHDFDNQVTLSGVLEINEESHENWWSAMTSDNCLEDELWVDQCSSMDVEFGSNEEFDDMLLWLWDDI*

>LW018VOUCH_Brunfelsia_pilosa_ MYBFL

MCEFVDLFFFFFFFNFFGCTIDTTITRECGLLHGFVYKGLSRCGKSCRLRWTNYLRPNLKRGKFTTEEDETIVKLQHSLGNRWALIASYLPGRTDNEIKNYWNSHLRRKIYTFSVNNNTSIETTVELAKKITNIADGINSESLRKRGRVSRSKAKKYNNNSTTTTFVSTLKTKSSCVGRSGRATRPNLSENFTIDREFLALPLFEETGLAIQQHECLVESAIGDPRNEEAEDNNQKDIDATPTVQEVCNGVLSLGEQRRKILDEHNNVEGPHYKNGSGETRLVQQPNYFLHDFDNQVSLSGVLEKNEVGENQWSTMISDHFLEDELWVDQCNSQDLEFGSIEECTTCCDDMLLWLWDDN*

>LW001A_Nierembergia_scoparia_ MYBFL

MGRAPCCEKVGLKRGRWSSQEDELLVKYMQANGEGSWRTLPKNAGLLRCGKSCRLRWINYLKPNLKRGKFTSQEDETIVKLQYSLGNRWSLISTYLPGRTDNEIKNYWNSHLRRRMYTFMLKNKHIEKTTLELPKRTIVVGKSLRKQGRVSLSKAKKYNNNSNNTTTYVSTLKIKSSCLGADGGGGATWSGGDYSVESHLSKKFTVDTGKVFHTYRYMH

>SS679A_Fabiana_imbricata_ MYBFL

MVRAPCCEKVGLKRGRWTAEEDDLLLKYIQANGEGLWKSLPKNAGLLRCGKSCRLRWTNYLRPNLKRCKFTSEEDETIVKLQCSLGNRWSLIASCLPGRTDNEIKNYWNSHLRRRIYTFRMKKKPIKTTAEVPKKTIVADGINCESLRKRGRVSRSKAKKYNSNTTTTATAYISSLKPKSSCGGAGSRATWSDGDSIEFHLSESFTIDTGNKFHTYKAYARCIFIFYP*

>LW010A_Calibrachoa_linoides_ MYBFL

MVRAPCCEKAGLKRGRWTAEEDELLLKYIQANGEGSWRSLPKSAGLLRCGKSCRLRWTNYLRPNLKRGKFTSEDETIVKLQCSLGNRWSLIASYLPGRTDNEIKNYWNSHLRRRIYTFRMKKKPIKTTAEVPKMTIVADGINCESLRKCGRVSRSKAKKYNHNTTTTATAYISSLKPKSSCVNVGAHSGATWSEGDSIEFDLSEIFTIDTAYLATLEGIGLDMQQHEHYAGSAIGKPRNVEREGNNEKHINATPEEVEVCHGMWSFEETEGKNQKQINATPEKQLEVCNGMWFFEEQGQQVLDEQIPNGPHETNAGDETVYLQQPNYCLHDFDNQVTLSGVLEINEESHENWWSAMTSDNCLEDELWVDQCSSMDVEFGSNEEFDDMLLWLWDDI*

>LW007A_Calibrachoa_heterophylla_ MYBFL

MVRAPCCEKAGLKRGRWTAEEDELLLKYIQANGEGSWRSLPKSAGLLRCGKSCRLRWTNYLRPNLKRGKFTSEDETIVKLQCSLGNRWSLIASYLPGRTDNEIKNYWNSHLRRRIYTFRMKKKPIKTTAEVPKMTIVADGINCESLRKCGRVSRSKAKKYNHNTTTTATAYISSLKPKSSCVNVGAHSGATWSEGDSIEFDLSEIFTIDTAYLATLEGIGLDMQQHDEHYAGSAIGKPRNVEREGNNEKHINATPEEEEVCHGMWSFEETEGKNQKQINATPEKQLEVCNGMWSFEETEGKNQKQINATPEKQLEVCNGMWSFEETEGKNQKQINATPEKQLEVCNGMWFFEEQEQQVLDEQIPNGPHETNAGDETVYLQQPNYCLHDFDNQVTLSGVLEINEESHENWWSAMTSDNCLEDELWVDQCSSMDVEFGSNEEFDDMLLWLWDDI*

>JN001A_Petunia_exserta_ MYBFL

EISFPHPAPPKAHSLYLIAVCHYVICTNVSKDSLAISLTEMVRAPCCEKVGIKRGRWTAEEDELLLKYIQANGEGSWRSLPKNAGLLRCGKSCRLRWTNYLRPNLKRGKFTSEEDETIFKLQCSLGNRWSLMASYLPGRTDNEIKNYWNSHLRRRIYTFGMKKKPIKTAAEMPNKTIVADGLNCESLKKRGRVSRSKAKKYNNNTTTTTTAYISTLKPKSQVLEPVVELYALKEIQLLIQVKSFIHIKAYFFYFPILPLITLSKMT*

>SS627B_Nierembergia_pulchella_ MYBFL

MWKELQIKMDKLLETKLEGKFTSQEDETIVKLQYSLGNRWSLISTYLPGRTYNEIKNYWNSHLRRRMYTFMLKNKHIEKTTLELPKRTIVAGKSLRKQGRVSLSKAKKYNNNSNTTSTYVSTLKIKSSCLGADGGGGATWSGGDYSVESHLSKKFTVDTELLTSPQLEEETRLPMQQNHATPENQEVCNGMWSFDKEGGHEQILLDHDDQHLQIIGPLEKNEGDVSVLLEKPNYCLNEFEEGKNWWSTMNISDNNFLEDELWVQDQCNFSLDMDFGSIEESTTYCDDDDDDDDMLLWLWNDI*

>SS604VOUCH_Nierembergia_linarifolia_ MYBFL

MGRAPCCEKVGLKRGRWSSQEDELLVKYIQANGEGSWRTLPKNAGLLRCGKSCRLRWINYLKPNLKRGKFTSQEDEIIVKLQYSLGNRWSLISTYLPGRTDNEIKNYWNSHLRRRMYTFMLKNKHIEKTTLELPKRTIIASKSLRKQGQVSRPKAKKYNNNNNTNTTYVSTLKIKSSCLGGDGGGGATWSGGDYSVESHLSKKFIVDTEFLTSPQLEKETRLAMQQNHATPENQEVCNGMWSFDKEGGHEQILLDHDDQHIKIIGPLERNEGDVSVLLEKPNYCLNEFEDGKNWWSTMNISDNNFLEDELWVQDHCNFSLDMDFGSIEESTTYCDDDDMLLWLWNDI*

>SS683B_Brunfelsia_australis_ MYBFL

MVRAPCCEKVGLKRGRWTAEEDELLVKYIQANGEGSWRSLPKNAGLSRCGKSCRLRWTNYLRPNLKRGKFTTEEDETIVKLQHSLGNRWALIASYLPGRTDNEIKNHWNSHLRRKIYTFSVNNNTSIKTTAELAKKITNIADDINSESLRKRGRLSRSKAKKYNNNSTTTTFVSTLKTKSSCVGRSGRATRPNSSENFTIDREFLALPLLEETGLAIQQHECPVESAIGDPRNEEAEDNNQKDINATPRVQEVCNGVLSLGEQRRKILDEHNNIEGPHYKNGSGETRLVQQPNYFLHDFDNQVSLSGVLEKNEVGENQWSTMISDHFLEDELWVDQCNRQDLEFGSIEECTTCCDDMLLWLWDDN*

>SS684A_Brunfelsia_americana_ MYBFL

MVKAPCCEKIGLKRGRWTAEEDELLLKYIQANGEGSWRFLPKNAGNNLSFLLSCSKPPRLINAGKNVTSTMHEAGFCWLEHNNVNKDMCEFVDLFFFFFFCGTIDTTITRECGLLHGFVYKGLSRCGKSCRLRWTNYLRPDLKRGKFTTEEDETIVKLQHSLGNRWALIASHLPGRTDNEIKNYWNSHLRRKIYTFRVNNSSSIKTTVELPKKITNVADGINSESLRKRERVSRSEAKKYNNNSTTTTFVSTLKPKSSCVGRSGRATRPKSSENFTIDKEFLALQLLEETGLAIQQHECPLESAIGDTRNEEAEDNSQKDNNATARVQEVCNGVWSFEEQRRKVLDEHNNTKGPHDKNGSDEATLVQQPNYCLHDFDNQVSLSGVLEMNEVGEN*

>SS626A_Fabiana_friesii_ MYBFL

MVRAACCEKVGLKRGRWTAEEDDLLLKYIQANGEGLWKSLPKNAGLLRCGKSCRLRWTNYLRPNLRRGKFTSEEDETIVKLQCSLGNRWSLIASCLPGRTDNEIKNYWNSHLRRRIYNFRMKKKPIKTTAEVPKKTIVADGINCEFLRKRGRVSRSKAKKYNSNTTTTATAYISSLKPKSSCVGAGSRATWSDGDSIEFHLSENFTIDTGNKFHTYKAYACRIFIFYP*

>LW026A_Fabiana_peckii_ MYBFL

MVRAPCCEKVGLKRGRWTAEEDDLLLKYIQANGEGLWKSLPKNAGLLRCGKSCRLRWTNYLRPNLKRGKFTSEEDETIVKLQCFLGNRWSLIASCLPGRTDNEIKNYWNSHLRRRIYTFRMKKKPIKTTAEVPKKTIVADGINCEFLRKRGRVSRSKAKKYNSNTTTTATAYISPA*

>SS624C_Fabiana_punensis_ MYBFL

LKYIQANGEGLWKSLPKHAGLLRCGKSCRLRWTNYLRPNLKRGKFTSEEDETIIKLHCSLGNRWSFIASCLPGRTDNEIKNYWNSHLRRKIYTFRMKKKPIKTTAEVPKKTIVADGINCEFLRKRGRVSRSKAKKYNSNTTTTATAYISSLKPKSSCVGAGSRATWSDGDSIEFHLSKFFTIDTETLALLEGKGQAIQQHDEHDAGSAIGKPRNEETEGKDQKDINATPEKQEVCNVAWSFEEQGQQVLDEHIPIGPHETNEGDETVHLQQTNYCLRDFENQVSLSGALEDNEGSRENWRSMNFDNFLEDELCVDQCSSLNLEFGSSEECDDMSLWLWDDN*

>LW015A_Brunfelsia_cuneifolia_ MYBFL

MAAELPGRTDNEIKNYWNSHLRRKIYTFSVNNNTSIETTVESAKKITNIADGINSESLRKRGRVSRSKAKKYNNNSTTTTFVSTLKTKSSCVGRSGRATRPNSSENFTIDREFLALPLFEETGLAIQQHECPVESAIGDPRIEEAEDNNQKDINATPRVQEVCNDVLSLGEQRRKILDEHNNIEGPHYKNGSGETRLVQQPNYFLHDFDNQVSLSGVLEKNEVGENQWSTMISDHFLEDELWVDQCNSQDLEFGSIEECTTCCDDMLLWLWDDN*

>LW020A_Petunia_inflata_ MYBFL

MVRAPCCEKVGIKRGRWTAEEDELLLKYIHANGEGSWRSLPKNAGLLRCGKSCRLRWTNYLRPNLKRGKFTSEEDETIVKLQCSLGNRWSLMASYLPGRTDNEIKNYWNSHLRRRIYTFRMKKKPIKTVAEVPNKTIVADGLNCESLKKRGRVSRSKAKKYNNNITTTTTAYISTLKPKSSCVGAGGGATCSEGDSIVDTGKKFQYT*

>Soltu.DM.05G005350.1_1_MYBFL

MGRFDKEGLKKGPWTPEEDQKLLSFIDKHGCGSWRALPAKAGLQRCGKSCRLRWINYLRPDIKRGKFSLQEERTIIQLHALLGNRWSAIAAYLPSRTDNEIKNYWNSRLKKRLTKMGIDPMTHKPNGEGSSKYVANLSHMAEWESARLEAEARLVQKSKIFFNNNNNSHNYNINPSTISQQLPYYQQLPCLDILKAWQMTSTKLPTINDISHAILRNNSKNKKLDSSIPSSTTNIFANNAPTTTKVGDDHQNLHDLSTINSCFEDDHLQTELPSFMQEFSGLFPEYTQNSTNGLQVDNTMGSCSGDFEDNKLLINWNDFPNYMVNSPIDCIN*

>LW014A_Petunia_altiplana_AN2

MSTSNASTSGVRKGAWTEEEDLLLRECIEKYGEGKWHLVPVRAGLNRCRKSCRLRWLNYLRPHIKRGDFSLDEVDLILRLHKLLGNRWSLIAGRLPGRTANDVKNYWNTHLRKKLIAPHDQKQESKNKAMKITENNIIKPRPRTFSRPAMNNFSCWNGKSCNKNTIDKNEGDTEIIKFSDENQKREESIDDGLQWWANLLANNIEIEELVSYNSPTLLHEETAPSVNAESSLTQEGGSGLSDFSVDIDDIWDLLG*

>JN042VOUCH_Petunia_secreta_AN2

MSTSNASTSGVRKGAWTEEEDLLLRECIEKYGEGKWHLVPVRAGLNRCRKSCRLRWLNYLRPHIKRGDFSLDEVELILRLHKLLGNRWSLIAGRLPGRTANDVKNYWNTHLRKKLIVPHDQKQESKTAMKITENNIIKPRPRTFSRPAMNHVSCWNGKSCNKNTIDKNEGDTEIIKFTDEKQKPEESIDDGLQWWANLLANNIEIKDLANGNSPTLLHEEIAPLVNIESNLMQEGESGLSDFSVDIDGIWDLLS*

>LW022A_Petunia_interior_AN2

MSTSNASTSGVRKGAWTEEEDLLLRECIDKYGEGKWHLVPVRAGLNRCRKSCRLRWLNYLRPHIKRGDFSLDEVDLILRLHKLLGNRWSLIAGRLPGRTANDVKNYWNTHLRKKLIAPHDQKQESKNKAMKITENNIIKPRPRTFSRPAMNNFSCWNGKRCNKNTIDKNEGDTEIIKFSDENQKREESIDDGLQWWANLLANNIEIEELVSYNSPTLLHEETAPSVNVESSLTQEGGSGLSDFSVDIDDIWDLLG*

>LW011VOUCH_Petunia_reitzii_AN2

MSTSNASTSGVRKGAWTEEEDLLLRECIEKYGEGKWHLVPVRAGLNRCRKSCRLRWLNYLRPHIKRGDFSEDEVDLILRLHKLLGNRWSLIAGRLPGRTANDVKNYWNTHLRKKLIAPHDQKQESKNKAMKITENNIIKPRPRTFSRPAMNNFSCWNGKSCNKNTIDKNEGDTEIIKFSDENQKREESIDDGLQWWANLLANNIEIEELVSYNSPTLLHEETAPSVNAESSLTQEGGSGLSDFSVDIDDIWDLLG*

>JN041A_Petunia_integrifolia_AN2

MSTSNASTSGVRKGAWTEEEDLLLRECIDKYGEGKWHLVPVRAGLNRCRKSCRLRWLNYLRPHIKRGDFSLDEVDLILRLHKLLGNRWSLIAGRLPGRTANDVKNYWNTHLRKKLIAPHDQKQESKNKAMKITENNIIKPRPRTFSRPAMNNFSCWNGKSCNKNTIDKNEGDTEIIKFSYENQKREESIDDGLQWWANLLANNIEIEELVSYNSPTLLHEETAPSVNVESSLTQEGGSGLSDFSVDIDDIWDLLG*

>SS605A_Petunia_axillaris_AN2

MNHVSCWNGKSCNKNTIDKNEGDTEIIKFTDEKQKPEESIDDGLQWWANLLANNIEIKDLANGNSPTLLHEEIAPLVNIESNLMQEGESGLSDFSVDIDGIWDLLS*

>LW023A_Petunia_correntina_AN2

MSTSNASTSGVRKGAWTEEEDLLLRECIEKYGEGKWHLVPVRAGLNRCRKSCRLRWLNYLRPHIKRGDFSLDEVDLILRLHKLLGNRWSLIAGRLPGRTANDVKNYWNTHLRKKLIAPHDQKQESKNKAMKITENNIIKPRPRTFSRPAMNHVSCWNGKSCNKNTIDKNEGDREIIKFSDEKQKPEESIDDGLQWWANLLANNIEIEELVSCNSPTLLHEETAPSVNAESSLTQEGGSGLSDFSVDIDDIWDLVS*

>JN049VOUCH_Petunia_bonjardensis_AN2

MKITENNIIKPRPRTFSRPAMNNFSCWNGKRCNKNTIDKNEGDTEIIKFSDENQKREETIDDGLQWWANLLANNIEIEELVSYNSPTLLHEETAPSVNVESSLTQEGGSGLSDFSVDIDDIWDLLG*

>LW017A_Petunia_scheideana_AN2

MKITENNIIKPRPRTFSRPAMNHVSCWNGKSCNKNTIDKNEGDREIIKFSDEKQKPEESIDDGLQWWANLLANNIEIEELVSCNSPTLLHEETAPSVNAESSLTQEGGSGLSDFSVDIDDIWDLVS*

>LW020A_Petunia_inflata_AN2

MKITENNIIKPRPRTFSRPAMNYFSCWNGKSCNKNTIDKNEGDTEIIKLSDEKQKPEESIDDGLQWWANLLANNIEIEELGSCNSPTLLHEETVPSVNAESSLTREGGSGLSDFSVDIDDIWDLVS*

>JG1003B_Calibrachoa_thymifolia_AN2

MMITSNASTSGVRKGAWTEEEDLLLRKCIEKYGEGKWHLVPVRAGLNRCRKSCRLRWLNYLRPHIKRGDFSLDEVDLILRLHKLLGNRWSLIAGRLPGRTANDVKNYWNTHVRKKLIAPHDQKEKSKNKAMKITENNIIKPRPRTFSRPAMNHVSCWNNKSCKENATDKNEGDREIIRFSDEKQKTEESIDDGLHWWANLLANSTEVDELSNGNSPTIFHEEIAPSVNVESNPMQGGGSGLSDFSADIDIWDLLS*

>LW012A_Calibrachoa_eglandulata_AN2

MYKNLMLVLCWFRWSLIAGRLPGRTANDVKNYWNTHVRKKLIAPHDQKEKSNKAMKISENNIIKPRPRTFSRPAMNHVSCWNNKSCKENATDKNEGDREIIRFSDEKQKTEESIDDGLHWWANLLANSTEVDELSNGNSPTIFHEEIAPSVNVESNPMQEGGSGLSDFSADIDIWDLLS*

>JG1090A_Calibrachoa_pubescens_AN2

MMITSNASTSGVRKGAWTEEEDLLLRKYIEKYGEGKWHLVPVRAGLNRCRKSCRLRWLNYLRPHIKRGDFSEDEVDLILRLHKLLGNRWSLIAGRLPGRTANDVKNYWNTHVRKKLIAPHDQKEKSKNKAMKITENNIIKPRPRTFSRPAMNHVSCWNNKSCKENATDKNEGDREIIRFRDEKQKTEESIDDGLHWWANLLANSTEVAELSNGNSLTIFHEEIAPSVDVESNPMQGGGSGLSDFSAGIDIWDLLS*

>JG1007VOUCH_Calibrachoa_longistyla_AN2

MMITSNASTSGVRKGAWTEEEDLLLRKCIEKYGKGKWHLVPVRAGLNRCRKSCRLRWLNYLRPHIKRGDFSLDEVDLILRLHKLLGNRWSLIAGRLPGRTANDVKNYWNTHVLKKLIAPHDQKEKSKNKAMKITENNIIKPRPRTFSRPAMNHVSCWNNKSCKENATDKNEGDREIIRFRDEKQKTEESIDDGLHWWANLLANSTEVAEFSNGNSPTIFHEEIAPSVDVESNPMQGGGSGLSDFSAGIDIWDLLS*

>LW009A_Calibrachoa_sendtneriana_AN2

MMITSNASTSGVRKGAWTEEEDLLLRKCIEKYGEGKWHLVPVRAGLNRCRKSCRLRWLNYLRPHIKRGDFSLDEVDLILRLHKLLGNRWSLIAGRLPGRTANDVKNYWNTHVRKKLIAPHDQKEKSKNKAMKITENNIIKPRPRTFSRPAMNHVSCWNNKSCKENATDKNEGDREIIRFSDEKQKTEESIDDGLHWWANLLANSTEVDELSNGNSPTIFHEEIAPSVNVESNPMQEGGSGLSDFSADIDIWDLLS*

>JN048VOUCH_Calibrachoa_sellowiana_AN2

MMITSNASTSGVRKGAWTEEEDLLLRKCIEKYGEGKWHLVPVRAGLNRCRKSCRLRWLNYLRPHIKRGDFSLDEVDLILRLHKLLGNRWSLIAGRLPGRTANDVKNYWNTHVRKKLIAPHDQKEKSKNKAMKITENNIIKPRPRTFSRPAMNHDSCWNNKSCKENATDKNEGDREIIRFSDEKQKTEESIDDGLHWWANLLANSTEVDELSNGNSPTIFHEEIAPSVNVESNPMQEGGSGLSDFSADIDIWDLLS*

>JN060VOUCH_Calibrachoa_irigangiana_AN2

MMITSNASTSGVRKGAWTEEEDLLLRKCIEKYGEGKWHLVPVRAGLNRCRKSCRLRWLNYLRPHIKRGDFSLDEVDLILRLHKLLGNRWSLIAGRLPGRTANDVKNYWNTHVRKKLIAPHDQKEKSKNKAMKITENNIIKPRPRTFSRPAMNHVSCWNNKSCKENATDKNEGDREIIRFSDEKQKREESIDDGLHWWANLLANSTEVDELSNGNSPTIFHEEIAPSVNVESNPMQEGGSGLSDFSADIDIWDLLS*

>SS663D_Calibrachoa_missionica_AN2

MMITSNASTSGVRKGAWTEEEDLLLRKCIEKYGEGKWHLVPVRAGLNRCRKSCRLRWLNYLRPHIKRGDFSLDEVDLILRLHKLLGNRWSLIAGRLPGRTANDVKNYWNTHVRKKLIAPHDQKEKSKNKAMKITENNIIKPRPRTFSRPAMNHVSCWNNKSCKENATDKNEGDREIIRFSDEKQKTEESIDDGLHWWANLLANSTEVDELSNGNSPTIFHEEIAPSVNVESNPMQEGGSGLSDFSADIDIWDLLS*

>LW010A_Calibrachoa_linoides_AN2

MMITSNASTSGVRKGAWTEEEDLLLRKCIEKYGEGKWHLVPVRAGLNRCRKSCRLRWLNYLRPHIKRGDFSLDEVDLILRLHKLLGNRWSLIAGRLPGRTANDVKNYWNTHVRKKLIAPHDQKEKSKNKAMKITENNIIKPRPRTFSRPAMNHVSCWNNKSCKENATDKNEGDREIIRFSDEKQKTEESIDDGLHWWANLLANSTEVDELSNGNSPTIFHEEIAPSVNVESNPMQEGGSGLSDFSADIDIWDLLS*

>JN055VOUCH_Calibrachoa_elegans_AN2

MMITSNASTSGVRKGAWTEEEDLLLRKCIEKYGEGKWHLVPVRAGLNRCRKSCRLRWLNYLRPHIKRGDFSLDEVDLILRLHKLLGNRWSLIAGRLPGRTANDVKNYWNTHVRKKLIAPHDQKEKSKNKAMKITENNIIKPRPRTFSRPAMNHVSCWNNKSCKENATDKNEGDREIIRFSDEKQKTEESIDDGLHWWANLLANSTEVDELSNGNSPTIFHEEIAPSVNVESNPMQEGGSGLSDFSADIDIWDLLS*

>JN081C_Calibrachoa_humilis_AN2

MMITSNASTSGVRKGAWTEEEDLLLRKCIEKYGEGKWHLVPVRAGLNRCRKSCRLRWLNYLRPHIKRGDFSLDEVDLILRLHKLLGNRWSLIAGRLPGRTANDVKNYWNTHVRKKLIAPHDQKEKSKNKAMKITENNIIKPRPRTFSRPAMNHVSCWNNKSCKENATDKNEGDREIISFSDEKQKTEESIDDGLHWWANLLANSTEVDELSNGNSPTIFHEEIAPSVNVESNPMQGGGSGLSDFSADIDIWDLLS*

>RD387B_Fabiana_bryoides_AN2

MITSNASTSGVRKGAWTEEEDLLLRKCIEKYGEGKWHLVPVRAGLNRCRKSCRLRWLNYPRPYIKRGDFSLAEVDLILRLHKLLGNRWSLIAGRLPGRTANDVKNYWNTHLRKKLIAPHDQKEKSKNKAMKITENIIKPRPRTFSMQAMNHISCWNDKSCNKNTIDKNEGDREIIRFSDEKQKTEESIDDGLQWWANLLANSTEVDELSNGNSPTLLHEEIAPSLNVESNPMQGGGSGLSDFSVDIDIWDLLS*

>JN059VOUCH_Calibrachoa_pygmaea_AN2

MINYNASTSGVRKGAWTEEEDLLLRKCINKYGEGKWHLVPVRADLNRCRKSCRLRWLNYLRPHIKRGDFSLDEVDLILRLHKLLGNRWSLIAGRLPGRTSNDVKNYWNTHLRKKLIAPYDQKEKSKNKAMKITENNIIKPRPRTFSRPAMNHISCWNNKNCKENAIDKNEDDTEIIRFSDEKQKNEESIDDGWWANLLANSTEVDELFNGNSPTLLHEEIAPCINVESNPMQEGGSGLSDFSIDIDIWDLLA*

>LW007A_Calibrachoa_heterophylla_AN2

MYKNLMLVLCWFRWSLIAGRLPGRTANDVKNYWNTHVRKKLIAPHDQKEKSKNKAMKITENNIIKPRPRTFSRPAMNHVSCWNNKSCKENATDKNEGDREIIRFSDEKQKTEESIDDGLHWWANLLANSTEVDELSNGNSPTIFHEEIAPSVNVESNPMQEGGSGLSDFSADIDIWDLLS*

>SS678A_Bouchetia_erecta_AN2

MNTCNDASSSGVRKGAWTEEEDLLLRKCIEKYGEGKWHLVPVRAGLNRCRKSCRLRWLNYLRPHIKRGDFSLDEVDLILRLHKLLGNRWSLIAGRLSRRTANDIKNYWNIHLRKKLIAPHEQRRLQQQQKSKNNNKTMKLSENTIIKPRPQTFVSRATSHASCWNNKNTIDKNEGDKQIKKFSDEKPKEDEMKDDDIQFWDKYLLANDNEIDELLAIENSSTLLHEEIVPVKNVENTTMQGGEICQRDFSVDIDIWDLLS*

>RD408B_Plowmania_nyctaginoides_AN2

MNTCNNNNTSSSGLRKGAWTEEEDLLLRKCIDKYGEGKWHLVPVRTGLNRCRKSCRLRWLNYLRPHIKRGDFSLDEVDLIWRLHKLLGNRWSLIAGRLPGRTANDVKNYWNTHLRKKLIAPHEQQQLQQQHEGKNKNKAMKIPENTIIKPRPRTFVSRATSHASCYYWNNKKTIDKNEGDKEIKKFSDEKPEEDEIIDDEIQLWAKYLLANDNEIDELLANEKSSSLSHEEIVPVINVENNIMQGEVGLRDFSVDIDIWDLLS*

>JN082A_Hunzikeria_texana_AN2

MNTCDNTSSSGVRKGAWTKEEDLSLRKCIDKYGEGKWHLVPVRAGLNRCRKSCRLRWMNYLRPHIKRGEFSLDEVDLILRMHKLIGNRWSLIAGRLPGRTSNDVKNYWNTHIKKKLIAPSEQQQQQYICEKKNKGMQVSENSIIKPRPHRTFVSNAASHVSCWNNNTTIDKNEANKEIKTFSDETPKEEETISDGFQWWANLLSNDYGIDEILPNENSSTLLHEEIIPLVNFENYPLQEGDIDLSDISVNIDIWDL*

>SS622B_Nierembergia_browallioides_AN2

ENYYYIIMSTCNNTSSSGVRKGAWTEEENLLLRKCIDKYGEGKWYLVPVRAGLNRCRKSCRLRWMNYLRPHIKRGDFSLDEVDLILRMHKLLGNRWSLIAGRLPGRTANDIKNYWNTHLQKKLIASHEQQQQQQQQKSKNKNRAMKISDNTIIKPRPRRTFIARATSHVSCWNNKNTIDKNEGDHKEVQKLSDDEKPNEEEMIDDGIQWWAKYLLANDDNYEIDDLLANESSSTLLHEEIVPLINAKNNTMQEGEICPSDFSVDIDIWDLLS*

>SS604VOUCH_Nierembergia_linarifolia_AN2

IYFRCHFENYYYIIIMSACNNTSSSGVRKGAWTEEENLLLRKCIDKYGEGKWHLVPIRAGLNRCRKSCRLRWVNYLRPHIKRGDFSLDEVDLILRMHKLLGNRWSLIAGRLPGRTANDIKNYWNTHLQKKLIASDHEQQQQQQQKSNNKNKAMKISNNTIIKPRPRTFIARATSHVSCWNNKNTIIDKNEGDHKEVQKLSDDEKPNEEEEMKDDGIQWWAKYLLANYDNYEIDHDLLANESSSSTLLHEGIVPLINAKNNNTMQEGEICPSDFSVDIDIWDLLC*

>SS650B_Nierembergia_calycina_AN2

MNSTKPSGVRKGAWTEEEDLLLRKCIHKYGEGKWHLVPLRAGLNRCRKSCRLRWLNYLRPHIKRGDFSLDEVDLILRLHKLLGNRWSLIAGRLPGRTANDIKNYWNTHLRKKLIDPHEQQLQQQQQKKSKDKNKTSKMSENTIIKPRPRTFVSSITSHIPGWNNKNTIDKNKGDKEIKRFSDEIKPKEEEMIDDGIEWWSKYLLANDNEIDGLLAHENLSTLFHEEIVPLVNVEKIMNNTMQEGEIGQSDFSVDSDIWDLLS*

>LW019A_Nierembergia_rivularis_AN2

MNTPSSSEVKKGAWTEEEDLLLRKCIHKYGEGKWHLVPVRAGLNRCRKSCRLRWLNYLRPHIKRGDFSLDEIDLILRLHKLLGNRWSLIAGRLPGRTANDIKNYWNTHLRKKLIDPHEQQQQQQQQHQKQQQQKSKDKNKTSKMSENTIIKPRPRTFVSRVTSHIPGWNNKNTIDKNKGDKEIKRFSDEIKPKEEEMIDDGIEWWTKYLLANDNEIDELLAHENLPTLVHEEIVPLVNVEKIMNNPMQEGEIGLSDFSVDSDIWDLLS*

> Soltu.DM.10G020840.1_AN2

MNTPMCASLGVRKGSWTEQEDFLLRKCIQIYGEGKWHLVPARAGLNRCRKSCRLRWLNYLRPHIKRGDFAPDEVDLILRLHKLLGNRWSLIAGRLPGRTANDVKNYWNTHFQKKLNIIAPPPPPRPRPNHHLQIKHKSITVNKNEIIRPQPRNFSNVKKNNSHWCNNKSMITNTLDKDDKRCKEIVVNISEKPTGENTSSIDDGVQWWTNLLENCNEIEEEVAVTNFEKTPTMLLHEEISPPLINGEGNSMQQGQSHDWDDFSTDIDLWNLLN
